# Suppression of autophagy promotes fibroblast activation in p53-deficient colorectal cancer cells

**DOI:** 10.1038/s41598-021-98865-1

**Published:** 2021-09-30

**Authors:** Takanori Inoue, Yoshito Hayashi, Yoshiki Tsujii, Shunsuke Yoshii, Akihiko Sakatani, Keiichi Kimura, Ryotaro Uema, Minoru Kato, Hirotsugu Saiki, Shinichiro Shinzaki, Hideki Iijima, Tetsuo Takehara

**Affiliations:** Department of Gastroenterology and Hepatology, Osaka University Graduate School of Medicine, Suita, Japan

**Keywords:** Cancer, Gastroenterology

## Abstract

Deficiency of p53 in cancer cells activates the transformation of normal tissue fibroblasts into carcinoma-associated fibroblasts; this promotes tumor progression through a variety of mechanisms in the tumor microenvironment. The role of autophagy in carcinoma-associated fibroblasts in tumor progression has not been elucidated. We aimed to clarify the significance of autophagy in fibroblasts, focusing on the *TP53* status in co-cultured human colorectal cancer cell lines (*TP53*-wild-type colon cancer, HCT116; *TP53*-mutant colon cancer, HT29; fibroblast, CCD-18Co) in vitro. Autophagy in fibroblasts was significantly suppressed in association with *ACTA2, CXCL12, TGFβ1, VEGFA, FGF2*, and *PDGFRA* mRNA levels, when co-cultured with p53-deficient HCT116^*sh p53*^ cells. Exosomes isolated from the culture media of HCT116^*sh p53*^ cells significantly suppressed autophagy in fibroblasts via inhibition of ATG2B. Exosomes derived from *TP53*-mutant HT29 cells also suppressed autophagy in fibroblasts. miR-4534, extracted from the exosomes of HCT116^*sh p53*^ cells, suppressed ATG2B in fibroblasts. In conclusion, a loss of p53 function in colon cancer cells promotes the activation of surrounding fibroblasts through the suppression of autophagy. Exosomal miRNAs derived from cancer cells may play a pivotal role in the suppression of autophagy.

## Introduction

Carcinoma-associated fibroblasts (CAFs) are major components of the tumor microenvironment and play pivotal roles in tumor progression^1–3^. Despite having originated from activated tissue fibroblasts, characteristics of CAFs vary significantly from fibroblasts in normal tissues^4^. The expressions of various fibrotic markers, growth factors, chemokines and cytokines are increased in CAFs than that in normal tissue fibroblasts; these factors may have tumorigenic effects^2^. Autophagy is a catabolic process that mediates the degradation of unnecessary or dysfunctional cellular components^5,6^. Different studies have reported the role of autophagy in cancer cells^7–9^. Recent reports have focused on the function of autophagy in both cancer and stromal cells^10–12^. Autophagy status of fibroblasts in the tumor stroma was reported to differ from that in extra-tumoral fibroblasts^12^. However, the significance of autophagy in the transition of normal tissue fibroblasts to CAFs during tumor progression and the mechanism of autophagy in CAFs remain unclear.

*TP53* is a tumor suppressor gene, and its mutational inactivation is frequently observed in many human cancers^13^. In colorectal cancer, *TP53* mutation is a common genetic abnormality that develops with the cancerization of the colon and is associated with chromosomal instability. p53 is a transcription factor that regulates the expression of genes associated with cell cycle arrest, apoptosis, and senescence. In addition, p53 has recently been suggested to modulate autophagy in cancer cells^14,15^. The *TP53* gene has a non-cell-autonomous function and can affect the surrounding cells in the tumor microenvironment, such as CAFs, by changing the secretion of a large number of proteins, production of reactive oxygen species, or profiles of miRNAs sequestered in the exosomes^16^. We have previously reported that the functional loss of p53 in colon cancer cells promoted the modification of tumor stroma and subsequent tumor growth through the above mechanisms^17,18^. However, to the best of our knowledge, the impact of p53 deficiency in cancer cells on the autophagy of co-existing fibroblasts has not been reported. Therefore, we aimed to clarify the significance of autophagy in the activation of fibroblasts, focusing on the *TP53* status of co-existing cancer cells in the tumor microenvironment.

## Results

### Inactivation of TP53 in cancer cells suppresses autophagy in fibroblasts

**Figure 1:**
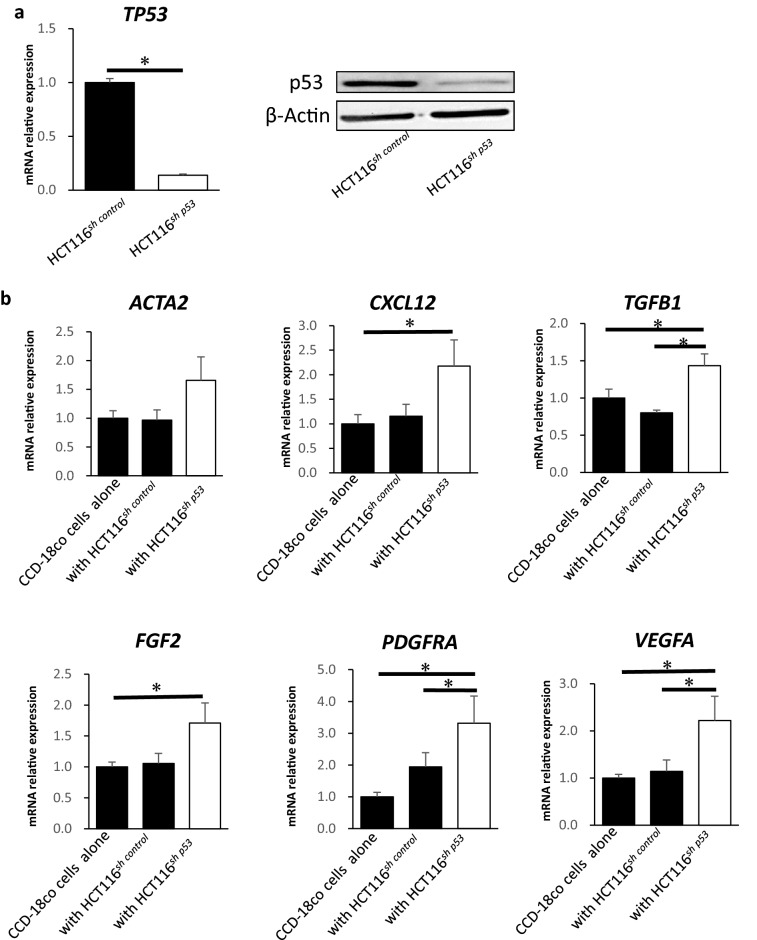

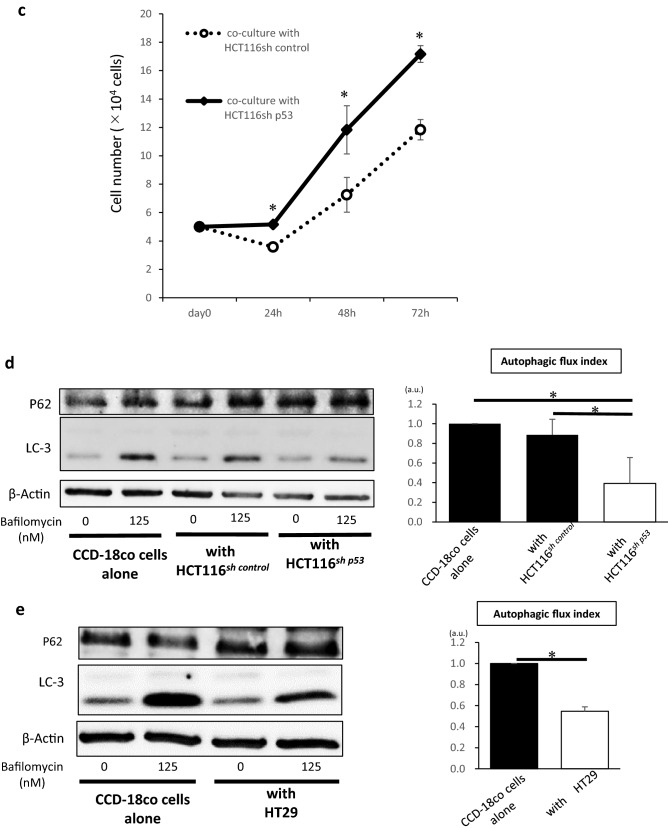
Inactivation of p53 in cancer cells activates fibroblasts and suppresses autophagy. (**a**) *TP53* mRNA expression analysis in HCT116^*sh p53*^ and HCT116^*sh control*^ cells using quantitative real-time PCR (qRT-PCR) (left; mean ± SD, *P < 0.05). The expression of p53 in HCT116^*sh p53*^ and HCT116^*sh control*^ cells using western blotting (right). (**b**) Relative expressions of *FGF2, PDGFRA, ACTA2, CXCL12, TGFβ1*, and *VEGFA* mRNAs in CCD-18Co cells co-cultured with or without HCT116^*sh p53*^ or HCT116^*sh control*^ cells analysed using qRT-PCR (*n* = 3, mean ± SD, *P < 0.05). (**c**) Cell numbers of CCD-18Co cells co-cultured with　HCT116^*sh p53*^ or HCT116^*sh control*^ cells for 24, 48, and 72 h. (*n* = 3, mean ± SD, *P < 0.05). (**d**) Western blotting and autophagic flux assays of CCD-18Co cells co-cultured with or without HCT116^*sh p53*^ or HCT116^*sh control*^ cells (*n* = 4, mean ± SD, *P < 0.05). (**e**) Western blotting and autophagic flux assays of CCD-18Co cells co-cultured with or without HT29 cells (*n* = 5, mean ± SD, *P < 0.05). Protein expression levels were measured using imageJ software 1.8.0_172 (https://imagej.nih.gov/).

The importance of autophagy function of fibroblasts has been reported previously^10–12^. Therefore, this study aimed to examine whether autophagy might play a role in the activation of fibroblasts. We intended to clarify the significance of autophagy in the activation of fibroblasts, while focusing on the *TP53* status. Thus, we examined activation and autophagy of CCD-18Co cells cocultured with HCT116^*sh control*^ cells and HCT116^*sh p53*^ cells in Fig. 1.

We used HCT116 cells which are human colon cancer cell lines with wild-type *TP53* expression.

To explore the significance of p53 functional deficiency in cancer cells, we generated *TP53*-deficient HCT116 cells (HCT116^*sh p53*^ cells) using shRNA for p53. We confirmed the suppression of *TP53* in HCT116 cells by qRT-PCR and western blotting analysis (Fig. 1a). The relative mRNA expression of *FGF2, PDGFRA*, *CXCL12, TGFβ1*, and *VEGFA*—the phenotypic markers of fibroblast activation—was higher in CCD-18Co cells co-cultured with HCT116^*sh p53*^ cells in Transwell than that in CCD-18Co cells co-cultured with HCT116^*sh control*^ cells and in those cultured alone (Fig. 1b). CCD-18Co cells co-cultured with HCT116^*sh p53*^ cells were found to have proliferated more than those co-cultured with HCT116^*sh control*^ cells (Fig. 1c). To clarify the effect of p53 deficiency in cancer cells on the autophagy of co-existing fibroblasts We evaluated autophagy in CCD-18Co cells co-cultured with HCT116^*sh control*^ and HCT116^*sh p53*^ cells by western blotting and autophagic flux assays. The autophagic flux was significantly suppressed in CCD-18Co cells co-cultured with HCT116^*sh p53*^ cells than that in CCD-18Co cells co-cultured with HCT116^*sh control*^ cells or cultured alone; p62(SQSTM1), which is a selective substrate for autophagy, accumulated in CCD-18Co cells co-cultured with HCT116^*sh p53*^ cells (Fig. 1d). These results suggest that autophagy in CCD-18Co cells co-cultured with HCT116^*sh p53*^ cells was suppressed than that in CCD-18Co cells co-cultured with HCT116^*sh control*^ cells or cultured alone. To confirm that the suppression of autophagy in fibroblasts was induced by the inactivation of p53 in cancer cells, we used HT29 cells that are colon cancer cells with mutations in *TP53*. Autophagy in CCD-18Co cells was significantly suppressed even when co-cultured with HT29 cells than that in CCD-18Co cells cultured alone, similar to the condition in CCD-18Co cells co-cultured with HCT116^*sh p53*^ cells (Fig. 1e). Therefore, autophagy in CCD-18Co cells was inhibited by some humoural factors secreted by cancer cells along with functional deficiency of p53.

### Suppression of autophagy induces activation of fibroblasts and accelerates growth in co-cultured cancer cells

**Figure 2:**
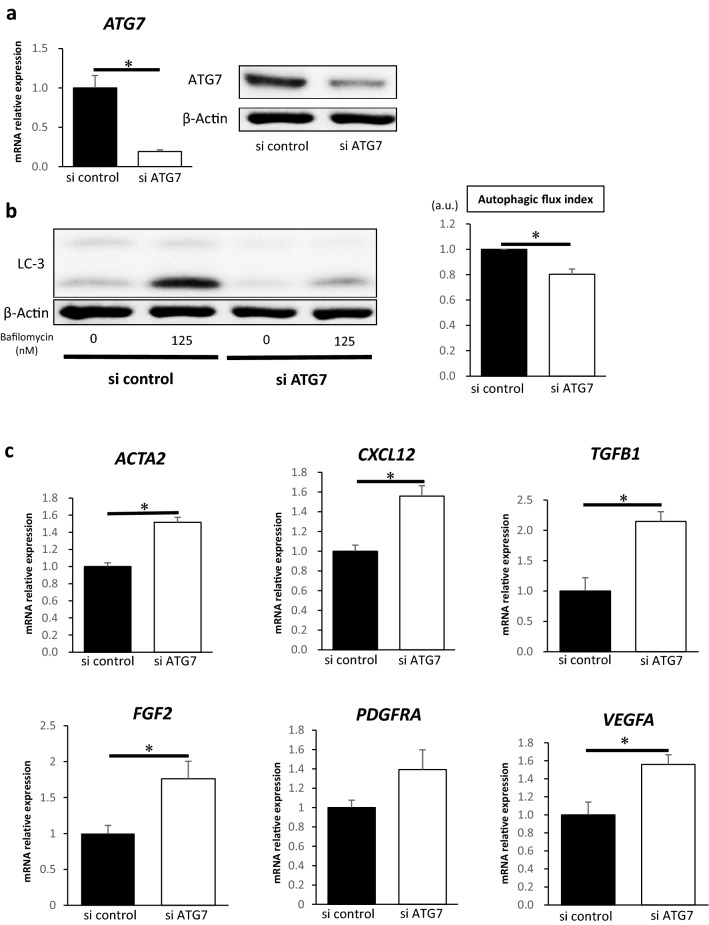

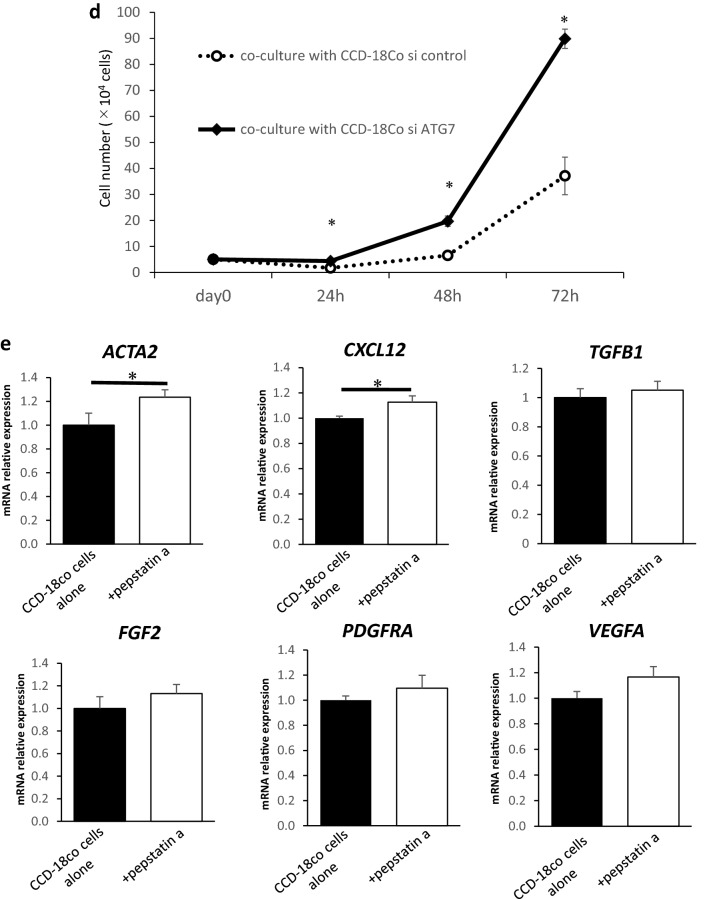
Suppression of autophagy activates fibroblasts. (**a**) *ATG7* mRNA expression in CCD-18Co cells transfected with *ATG7* or control siRNA analysed using quantitative real-time PCR (qRT-PCR) (left; *n* = 3, mean ± SD, *P < 0.05). The expression of ATG7 protein in CCD-18Co cells transfected with *ATG7* or control siRNA analysed using western blotting (right). (**b**) Western blotting and autophagic flux assays in CCD-18Co cells transfected with ATG7 or control siRNA (*n* = 4, mean ± SD, *P < 0.05). (**c**) Relative expressions of *ACTA2, CXCL12, TGFβ1*, *FGF2, PDGFRA* and *VEGFA* mRNA in CCD-18Co cells transfected with *ATG7* or control siRNA analysed using qRT-PCR (*n* = 3, mean ± SD, *P < 0.05). (**d**) Cell numbers of HCT116 cells co-cultured with CCD-18Co cells transfected with *ATG7* or control siRNA for 24, 48 and 72 h. (*n* = 3, mean ± SD, *P < 0.05). (**e**) Relative expressions of *ACTA2, CXCL12, TGFβ1, FGF2, PDGFRA* and *VEGFA* mRNA in CCD-18Co cells treated with pepstatin or control (*n* = 3, mean ± SD, *P < 0.05). Protein expression levels were measured using imageJ software 1.8.0_172 (https://imagej.nih.gov/).

Next, we examined whether the suppression of autophagy in fibroblasts can affect their activation. We suppressed autophagy in CCD-18Co cells using siRNA for *ATG7* (a major autophagy-related protein) and pepstatin (an autophagy inhibitor). We confirmed the inhibition of *ATG7* in CCD-18Co cells transfected with *ATG7* siRNA using qRT-PCR and western blotting (Fig. 2a). Transfection of *ATG7* siRNA significantly suppressed the autophagic flux in CCD-18Co cells than that of control siRNA (Fig. 2b). The relative expression levels of *ACTA2*, *CXCL12*, *FGF2*, *TGFβ1*, and *VEGFA* mRNA were high in CCD-18Co cells transfected with *ATG7* siRNA than in those with control siRNA (Fig. 2c). HCT116 cells co-cultured with CCD-18Co cells transfected with *ATG7* siRNA proliferated more than those co-cultured with CCD-18Co cells transfected with control siRNA (Fig. 2d). Moreover, to inhibit autophagy in fibroblasts in another way and observe the activation, we used the autophagy inhibitor pepstatinA. when pepstatin A was added to CCD-18Co cells, the relative expression levels of *ACTA2* and *CXCL12* mRNA increased than that in control cells (Fig. 2e). These results suggest that suppression of autophagy in fibroblasts may lead to their activation and promote fibroblast-mediated cancer cell proliferation.

### Exosomes derived from TP53-inactivated cancer cells suppress autophagy in fibroblasts

**Figure 3:**
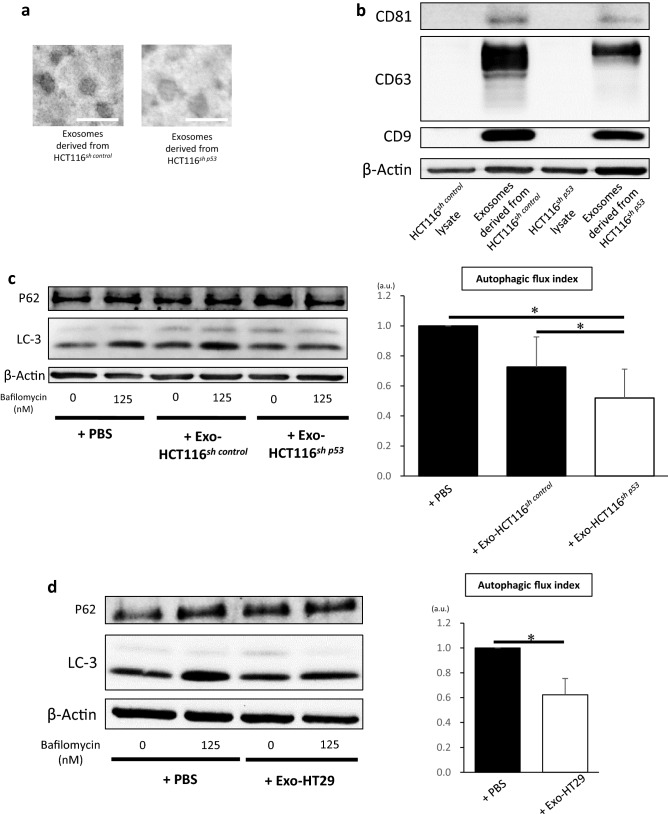
Exosomes derived from *TP53*-inactivated cancer cells suppress autophagy in fibroblasts. (**a**) Representative transmission electron microscopy images of exosomes isolated from culture media of HCT116^*sh control*^ or HCT116^*sh p53*^ cells. Scale bar: 100 nm. (**b**) Western blotting analysis of CD9, CD63, and CD81 in exosomes from HCT116^*sh control*^ or HCT116^*sh p53*^ cells. (**c**) Western blotting and autophagic flux assays of CCD-18Co cells cultured with or without exosomes derived from HCT116^*sh p53*^ (+ Exo-HCT116^*sh p53*^) or HCT116^*sh control*^ cells (+ Exo-HCT116^*sh control*^) (*n* = 5, mean ± SD, *P < 0.05). (**d**) Western blotting and autophagic flux assays of CCD-18Co cells cultured with (+ Exo-HT29) or without (PBS) exosomes derived from HT29 cells (*n* = 5, mean ± SD, *P < 0.05). Protein expression levels were measured using imageJ software 1.8.0_172 (https://imagej.nih.gov/).

Recently, p53 inactivation in cancer cells was reported to affect the surrounding stromal cells by modifying the secretion of miRNAs sequestered in exosomes^18^. We focused on the role of exosomes derived from cancer cells in the autophagy of fibroblasts in the tumor microenvironment. We isolated exosomes from the culture media of HCT116^*sh control*^ and HCT116^*sh p53*^ cells (Fig. 3a). Protein expression in the isolated exosomes was confirmed using western blotting with representative exosome markers, such as CD9, CD63, and CD81 (Fig. 3b). To examine whether exosomes derived from cancer cells were involved in the autophagy of fibroblasts, we observed the autophagic flux in CCD-18Co cells after the addition of exosomes derived from HCT116^*sh control*^ or HCT116^*sh p53*^ cells. The autophagic flux in CCD-18Co cells was significantly suppressed and accumulation of p62 was enhanced with the addition of exosomes from HCT116^*sh p53*^ cells than that of exosomes from HCT116^*sh control*^ cells and in control CCD-18Co cells (Fig. 3c). In addition, we stimulated CCD-18Co cells with exosomes derived from HT29 cells and examined their autophagic flux. The autophagic flux was significantly suppressed and accumulation of p62 was enhanced in CCD-18Co cells cultured with exosomes from HT29 cells than in CCD-18Co cells cultured in control PBS (Fig. 3d). These results suggest that exosomes derived from p53-deficient cancer cells may be involved in the suppression of autophagy in fibroblasts.

### Suppression of ATG2B activates fibroblasts via inhibition of autophagy

**Figure 4:**
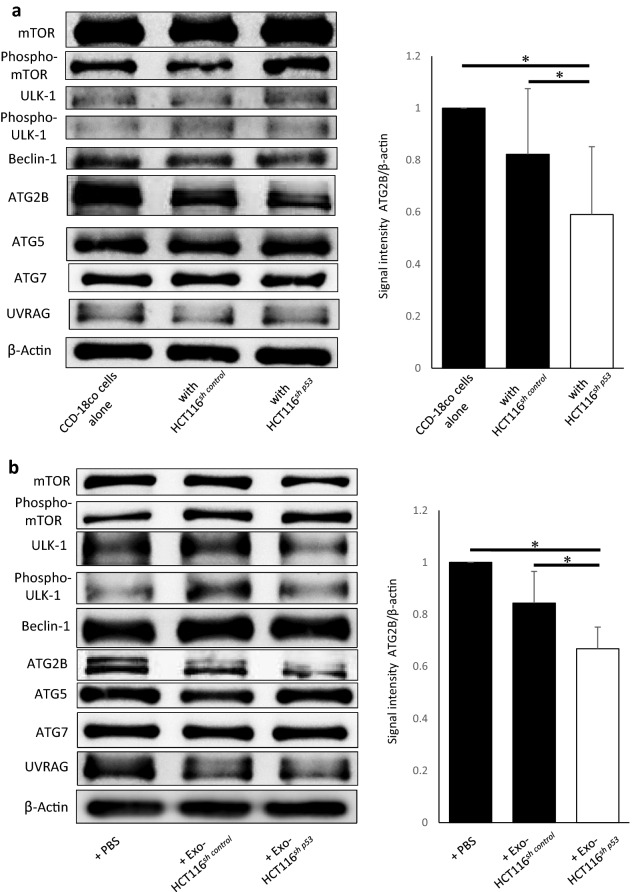

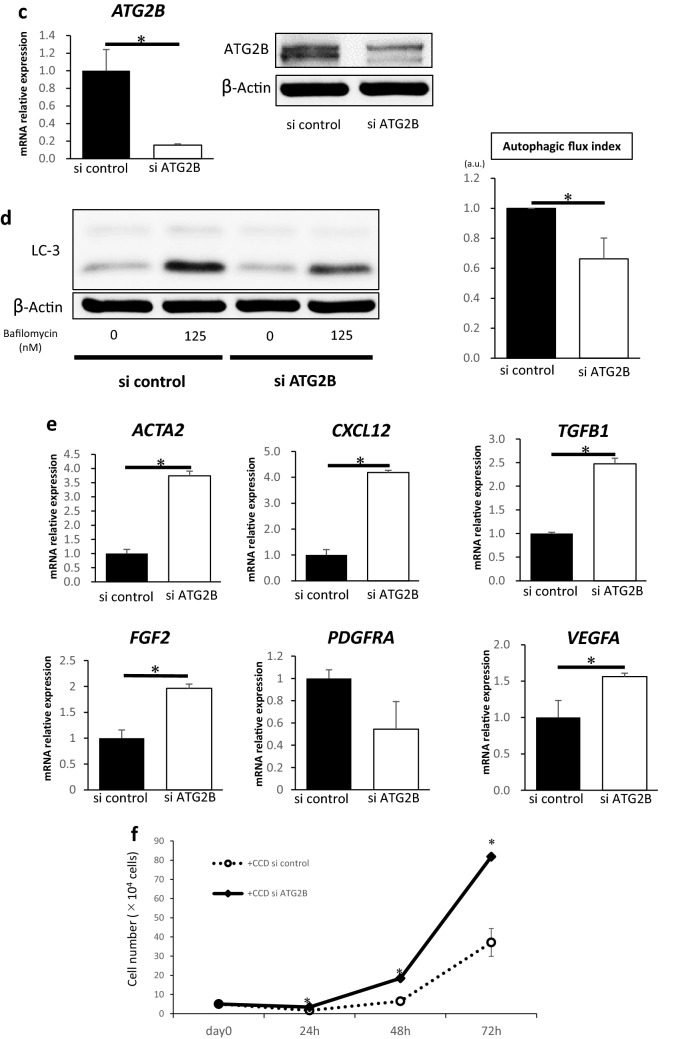
Suppression of ATG2B activates fibroblasts. (**a**) Western blotting analysis of autophagy-related proteins in CCD-18Co cells co-cultured with HCT116^*sh control*^ or HCT116^*sh p53*^ cells. Quantification of ATG2B signal intensity (*n* = 5, mean ± SD, *P < 0.05). (**b**) Western blotting analysis of autophagy-related proteins in CCD-18Co cells cultured with or without exosomes derived from HCT116^*sh p53*^ or HCT116^*sh control*^ cells. Quantification of ATG2B signal intensity (*n* = 5, mean ± SD, *P < 0.05). (**c**) *ATG2B* mRNA expression in CCD-18Co cells transfected with *ATG2B* or control siRNA analysed using quantitative real-time PCR (qRT-PCR) (left; *n* = 3, mean ± SD, *P < 0.05). The expression of ATG2B in CCD-18Co cells transfected with *ATG2B* or control siRNA analysed using western blotting (right). (**d**) Western blotting and autophagic flux assay in CCD-18Co cells transfected with *ATG2B* or control siRNA (*n* = 4, mean ± SD, *P < 0.05). (**e**) Relative expressions of *ACTA2, CXCL12, TGFβ1*, *FGF2, RDGFRA* and *VEGFA* mRNA in CCD-18Co cells transfected with *ATG2B* or control siRNA analysed using qRT-PCR (*n* = 3, mean ± SD, *P < 0.05). (**f**) Cell numbers of HCT116 cells co-cultured with CCD-18Co cells transfected with *ATG2B* or control siRNA for 24, 48, and 72 h. (*n* = 3, mean ± SD, *P < 0.05). Protein expression levels were measured using imageJ software 1.8.0_172 (https://imagej.nih.gov/).

To explore the mechanism of suppression of autophagy in fibroblasts associated with p53 inactivation in cancer cells, we examined the expression of representative autophagy-related proteins in CCD-18Co cells co-cultured with HCT116^*sh control*^ or HCT116^*sh p53*^ cells using western blotting. Among these autophagy-related proteins, the expression of ATG2B in CCD-18Co cells co-cultured with HCT116^*sh p53*^ cells was significantly suppressed than that in CCD-18Co cells co-cultured with HCT116^*sh control*^ cells or CCD-18Co cells cultured alone (Fig. 4a). In addition, the expression level of ATG2B protein in CCD-18Co cells cultured with exosomes derived from HCT116^*sh p53*^ cells was suppressed than that in CCD-18Co cells cultured with exosomes derived from HCT116^*sh control*^ cells and control CCD-18Co cells (Fig. 4b). Therefore, we hypothesized that the suppression of autophagy and subsequent activation of fibroblasts by HCT116^*sh p53*^ cells may be induced through ATG2B suppression. To determine the function of ATG2B in the activation of fibroblasts, we suppressed the expression of *ATG2B* in CCD-18Co cells using siRNA. We confirmed the inhibition of *ATG2B* in CCD-18Co cells by qRT-PCR and western blotting analysis (Fig. 4c). Inhibition of *ATG2B* suppressed autophagic flux in CCD-18Co cells than expression in control cells (Fig. 4d). The relative expression levels of *ACTA2*, *CXCL12*, *TGFβ1*, *FGF2* and *VEGFA* mRNA significantly increased in CCD-18Co cells with *ATG2B* suppression than in control CCD-18Co cells (Fig. 4e). We further investigated the effect of *ATG2B* suppression in CCD-18Co cells on the proliferation of co-existing cancer cells. The proliferation of HCT116 cells co-cultured with CCD-18Co cells with *ATG2B* suppression was significantly higher than that of HCT116 cells co-cultured with control CCD-18Co cells (Fig. 4f). These results suggest that the suppression of *ATG2B* in p53-deficient cancer cells can activate fibroblasts and accelerate fibroblast-mediated cancer cell proliferation.

### miR-4534 suppresses ATG2B expression in fibroblasts

**Figure 5:**
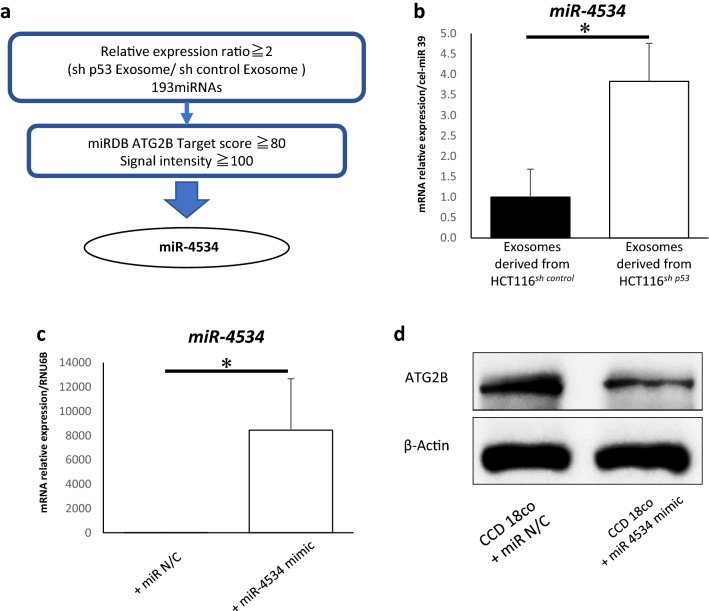

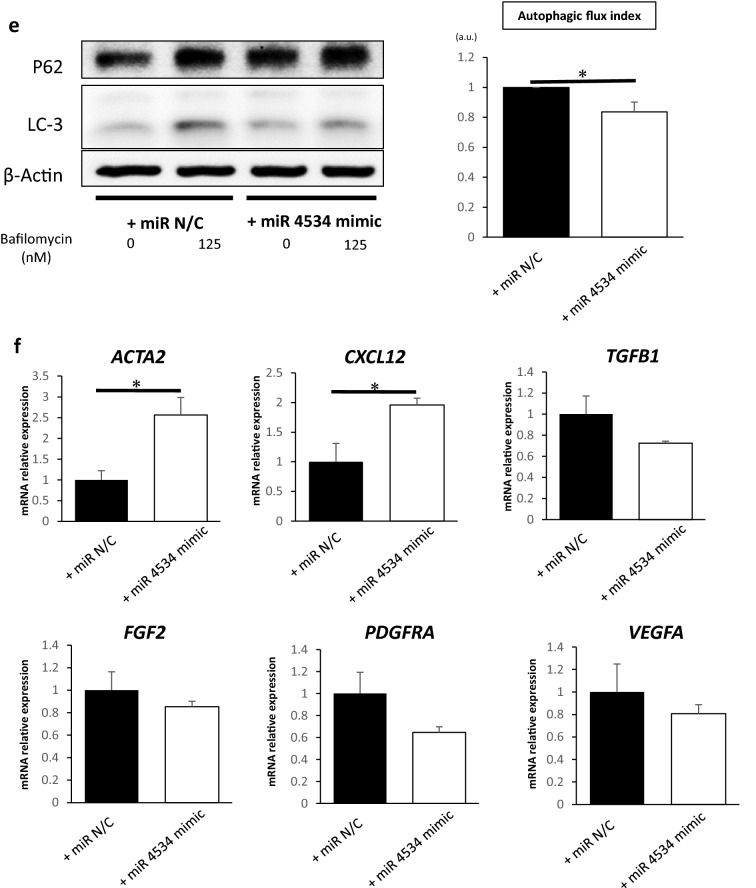
miR-4534 inhibits ATG2B expression in fibroblasts. (**a**) Strategy for identifying miR-4534 using microarray analysis and miRDB. (**b**) The relative expression levels of miR-4534 in exosomes derived from HCT116^*sh control*^ and HCT116^*sh p53*^ cells analysed using quantitative real-time PCR (qRT-PCR) (*n* = 4, mean ± SD, *P < 0.05). (**c**) Relative expressions of miR-4534 in CCD-18Co cells transfected with or without miR-4534 mimics analysed using qRT-PCR (*n* = 5, mean ± SD, *P < 0.05). (**d**) Western blotting analysis of ATG2B in CCD-18Co cells transfected with or without miR-4534 mimics. (**e**) Western blotting and autophagic flux assays in CCD-18Co cells transfected with or without miR-4534 mimics (*n* = 4, mean ± SD, *P < 0.05). (**f**) Relative expressions of *FGF2, PDGFA, ACTA2, CXCL12, TGFβ1*, and *VEGFA* mRNAs in CCD-18Co cells transfected with or without miR-4534 mimics analysed using qRT-PCR (*n* = 3, mean ± SD, *P < 0.05). Protein expression levels were measured using imageJ software 1.8.0_172 (https://imagej.nih.gov/).

Finally, we attempted to identify exosomal miRNAs that regulate *ATG2B* expression in fibroblasts. Our previous study compared the miRNA profiles in HCT116^*sh p53*^ and HCT116^*sh control*^ exosomes using miRNA microarrays, as detailed in the Gene Expression Omnibus database of the National Center for Biotechnology Information (GSE120013; https://www.ncbi.nlm.nih.gov/)^18^. We determined the miRNAs targeting *ATG2B* using the microarray dataset, based on their expression levels in HCT116^*sh p53*^ and HCT116^*sh control*^ exosomes and their probability of targeting *ATG2B* based on their sequences. miR-4534 was identified as the top de-regulated microRNA with a signal intensity of > 100 in HCT116^*sh p53*^ exosomes, an expression ratio (expression in HCT116^*sh p53*^ exosomes / expression in HCT116^*sh control*^ exosomes) > 2, and a target score > 80 as calculated in the miRDB (http://mirdb.org/)^19,20^ (Fig. 5a).

We compared the levels of miR-4534 expression in HCT116^*sh p53*^ and HCT116^*sh control*^ exosomes using qRT-PCR with syn-cel-miR-39 as the external control. miR-4534 expression in HCT116^*sh p53*^ exosomes was significantly higher than that in HCT116^*sh control*^ exosomes (Fig. 5b). We then over-expressed miR-4534 in CCD-18Co cells using the miRNA mimic (Fig. 5c). ATG2B expression in CCD-18Co cells was suppressed by the over-expression of miR-4534 than that by control miRNA (Fig. 5d). We examined the autophagy and activation of CCD-18co cells transfected with miR4534 mimic. We found that the autophagy of CCD-18co cells transfected with miR4534 mimic was more suppressed compared to that of CCD-18co cells transfected with miR control (Fig. 5e). The relative expression levels of *ACTA2* and *CXCL12* mRNA in CCD-18Co cells transfected with miR4534 mimic were higher than with that of CCD-18co cells transfected miR control (Fig. 5f).

## Discussion

In the present study, we showed that p53 deficiency in colon cancer cells suppressed autophagy and promoted subsequent activation of fibroblasts. Suppression of the autophagy-related protein, ATG2B*,* by exosomes derived from *TP53*-deficient colon cancer cells suppressed autophagy in fibroblasts. Furthermore, we also identified specific miRNAs in exosomes derived from *TP53*-deficient cancer cells that can suppress expression of ATG2B in fibroblasts.

The role of autophagy in tumor progression is still controversial^21–25^, particularly in the tumor stromal cells. It has been reported that autophagy in tumor stroma promotes tumor growth by supplying nutrients, including amino acids^11,26^. Another report also described the pro-tumorigenic effect of autophagy in CAFs by protecting against oxidative stress^10^; therefore, autophagy in CAFs promotes tumor growth. However, some studies have reported that the suppression of autophagy in fibroblasts can induce their activation^27^. For instance, myofibroblast differentiation in the pulmonary fibroblasts was promoted by suppression of autophagy and contributed to the pathogenesis of chronic obstructive pulmonary disease^28^. The present study showed that suppression of autophagy in colon fibroblasts co-existing with *TP53*-deficient colon cancer cells can induce activation of fibroblasts. Autophagy in tumor stromal fibroblasts may play diverse roles in different phases of tumor progression, such as in intratumoral nutrient supply or myofibroblast differentiation during a desmoplastic reaction. We believe that the suppression of autophagy in fibroblasts induced by cancer cells may be involved in their activation; fibroblast activation plays a pivotal role in the transition of normal tissue fibroblasts into CAFs and formation of tumor stroma. CAFs have higher expression of activation markers and tumor growth factors than normal fibroblasts^29^; our study showed that normal fibroblasts with suppressed autophagy had acquired properties similar to that of CAFs. This is the first report validating the association between the suppression of autophagy in colon fibroblasts and activation and transition of normal tissue fibroblasts into CAFs.

Normal tissue fibroblasts can acquire CAF-like phenotypes in response to soluble secretions from cancer cells, including exosomes^18,30,31^. Exosomes communicate information on myriad proteins, mRNAs and miRNAs between secreting and recipient cells, and hence, have an impact on the expression of various genes in recipient cells that governs the conversion of normal fibroblasts into CAFs^18,31^. Many target genes and related factors, such as *TP53,* sphingosine kinase 1, E-cadherin, TGFβ1, and senescence are involved in the activation of fibroblasts induced by cancer cell-derived exosomes^18,31–35^. CAFs have a heterogeneous cell population with varying origins, phenotypes, and functions in the tumor stroma^29^. Different processes are associated with the transition of normal tissue fibroblasts into CAFs; herein, we focused on the significance of autophagy in activated fibroblasts and identified a specific miRNA in exosomes that can suppress autophagy and induce fibroblast activation with CAF-like phenotypes. Although the detailed mechanisms of miRNA sorting into exosomes have not been elucidated, several reports have indicated that *TP53* expression in donor cancer cells can modify their inherent exosomal miRNA profiles and affect gene expression in surrounding cells^18,36^. After analysis of the miRNAs sorted into exosomes in donor cancer cells with p53 deficiency, we discovered that miR-4534 suppressed ATG2B and activated fibroblasts via suppression of autophagy.

A previous report indicated that miR-4534 was over-expressed in prostate cancer tissues and showed oncogenic effects by downregulating the tumor suppressor gene, *PTEN*^37^; however, most of its functions remain unclear. ATG2B is an autophagy-related protein that can regulate the transfer of lipids for autophagosome formation^38,39^. The present report demonstrates a novel and important function of miR-4534 as a regulator of autophagy via suppression of ATG2B in the tumor stroma during colorectal tumorigenesis. To reveal the clinical usefulness of miR-4534, a better understanding of its roles and further research are required.

In conclusion, loss of p53 function in colon cancer cells may promote the activation of surrounding fibroblasts through the suppression of autophagy. miR-4534 in the exosomes of *TP53*-deficient cancer cells activated fibroblasts by suppressing ATG2B. Therefore, we propose that the suppression of autophagy by specific miRNAs in exosomes can play an important role in the transition of normal tissue fibroblasts into CAFs and subsequent stroma-mediated tumor growth.

## Materials and methods

### Cell culture

Human colon cancer cell lines—HCT116 with wild-type *TP53* expression, and HT29 with *TP53* mutation—were cultured in Dulbecco’s modified Eagle’s medium (DMEM) (Sigma-Aldrich, St. Louis, MO, USA) supplemented with 10% fetal bovine serum. Non-transformed human colon fibroblasts (CCD-18Co) were cultured in Eagle’s minimum essential medium (EMEM) (ATCC, Manassas, VA, USA) supplemented with 10% fetal bovine serum. All cell lines were obtained from the American Type Culture Collection (ATCC); the cell lines were used within < 6 months after purchase, and were authenticated to verify their identity and the absence of cross-contamination (National Institute of Biomedical Innovation, Osaka, Japan). HCT116 and CCD-18Co cell lines were last authenticated on 23 February 2015, and HT29 cell line was last authenticated on 9 August 2018. All cell lines were used between 3 and 9 passages in this study.

### RNA interference

Lentiviral GFP-IRES-shRNA vectors against *TP53* (RHS4430-101161166; 101162286; 101168779; 99365289) were obtained from Thermo Fisher Scientific (Waltham, MA, USA), and HCT116^*sh control*^ and HCT116^*sh p53*^ cells were generated. Cells were cultured with 2 µg/ml puromycin after colony selection to maintain stable shRNA expression. The cultured fibroblast cell lines were transfected with small interfering RNAs (siRNAs) against the autophagy genes, *ATG2B* and *ATG7* (Invitrogen, Carlsbad, CA, USA), using the transfection reagent, Lipofectamine RNAiMAX (Invitrogen, 13778150), as described in the manufacturer’s instructions.

### Exosome isolation

Cancer cells (HCT116 or HT29) were first washed with phosphate-buffered saline (PBS), and then fresh serum-free DMEM was added. After incubation for 48 h, the conditioned medium was collected and centrifuged. Centrifugation was performed in three steps: first at 300×*g* for 10 min, then at 2000×*g* for 10 min to remove the cells, and subsequently at 10,000×*g* for 30 min. The extracellular vesicles (EVs) were separated by ultracentrifugation at 100,000×*g* for 70 min, and were washed by suspending in PBS. EVs were then ultracentrifuged again at 100,000×*g* for 70 min. The final pellet was re-suspended in 100 μL of PBS and collected as exosomes. Protein concentration of the purified exosomes was quantified with bicinchoninic acid protein assay kit (Thermo Fisher Scientific). Morphology of the exosomes was observed using transmission electron microscopy (Hitachi High-Technologies Corporation, Tokyo, Japan, H-7650) after preparation using the exosome-TEM-easy kit (101Bio, Palo Alto, CA, USA), according to the manufacturer’s instructions. Exosomes derived from cancer cells were added to fibroblasts at a concentration of 100 µg/mL.

### Co-culture experiments

We co-cultured the colon fibroblast (CCD-18Co cells) and cancer cells (HCT116^*sh control*^, HCT116^*sh p53*^, and HT29 cells) using Transwell inserts with 0.4 μm pore size (Corning, NY, USA). Fibroblasts or cancer cells were seeded in 6- or 12-well plates, and an equal amount of the other cell type was seeded in Transwell inserts. The culture media in the plates and Transwell inserts were changed to EMEM after 24 h, and the Transwell inserts were placed on companion plates. Further assays were performed after co-culturing the cells for 48–72 h.

### Cell proliferation and viability

We analyzed cell proliferation in 12-well plates (5 × 10^4^ cells per well). Co-cultured cells were counted using c-chips (NanoEntek, Seoul, Korea) at 24, 48 and 72 h respectively.

### Quantitative real-time polymerase chain reaction (qRT-PCR)

Total RNA was extracted from cell lines using the RNeasy kit (QIAGEN, Tokyo, Japan). Complementary DNA was synthesized from 1 µg of total RNA using the ReverTra Ace qPCR RT Kit (Toyobo, Osaka, Japan). The mRNA expression of genes was quantified using the TaqMan gene expression assay (Applied Biosystems, Foster City, CA). The expression of genes was normalized to that of *B2M* (beta-2-microglobulin).

We used miRNA-specific primers (TaqMan MicroRNA Assays, Applied Biosystems) for miRNA reverse transcription, and TaqMan gene expression assay for miRNA expression quantification. The expression data were normalized to *RNU6B* expression for cellular miRNAs and syn-cel-miR-39 spike-in (QIAGEN, Tokyo, Japan) expression for exosomal miRNAs.

### Western blotting analysis

Cultured cells were lysed with a lysis buffer (1% NP-40, 0.5% sodium deoxycholate, 0.1% sodium dodecyl sulfate and 1× protein inhibitor cocktail (Nacalai Tesque, Kyoto, Japan), phosphate-buffered saline; pH 7.4). After incubation on ice for 15 min, the lysate was centrifuged at 10,000*g* for 15 min at 4 °C. The protein content of the supernatants was determined using a bicinchoninic acid protein assay kit (Pierce, Rockford, IL). Equal amounts of protein were separated by sodium dodecyl sulphate–polyacrylamide (SDS) gel electrophoresis and transferred to polyvinylidene difluoride membranes (Bio-Rad, Hercules, CA, USA). We cut the membrane before hybridisation with antibodies. Protein expression levels were measured using imageJ software 1.8.0_172 (https://imagej.nih.gov/). All full length data of western blots are in Supplementary Information.

### Autophagic flux assay

We used bafilomycin A1 (BioViotica, Dransfeld, Germany), an autophagic flux inhibitor, prepared at 125 nM concentration in ethanol. Cells were treated with bafilomycin A1 for 2 h before protein extraction. The autophagic flux index was calculated using the following method: autophagy flux index = (LC3-II expression levels with bafilomycin A1)/(LC3-II expression levels without bafilomycin A1). LC3-II expression levels were normalized to beta-actin expression levels. In each experiment, the autophagic flux index of the control group was normalized to 1.

### MicroRNA expression analysis

We used miRNA mimics (TaqMan MicroRNA Assays, Applied Biosystems) to over-express miRNAs in CCD-18Co cells. We then evaluated the effect of this over-expression on *ATG2B* expression using qRT-PCR, as described above.

### Statistical analysis

Comparisons between groups were performed using student’s *t*-test. P < 0.05 was considered statistically significant.

## Supplementary Information

Supplementary Information 1.

Supplementary Information 2.
