# Supplementary material for: Suppression of autophagy promotes fibroblast activation in p53-deficient colorectal cancer cells
Source: Sci Rep. 2021 Sep 30;11:19524. doi: 10.1038/s41598-021-98865-1 (PMC8484348; doi:10.1038/s41598-021-98865-1)
Supplement: Supplementary file 1 — Supplementary Information 1. [file 41598_2021_98865_MOESM1_ESM.pdf]

Figure S1

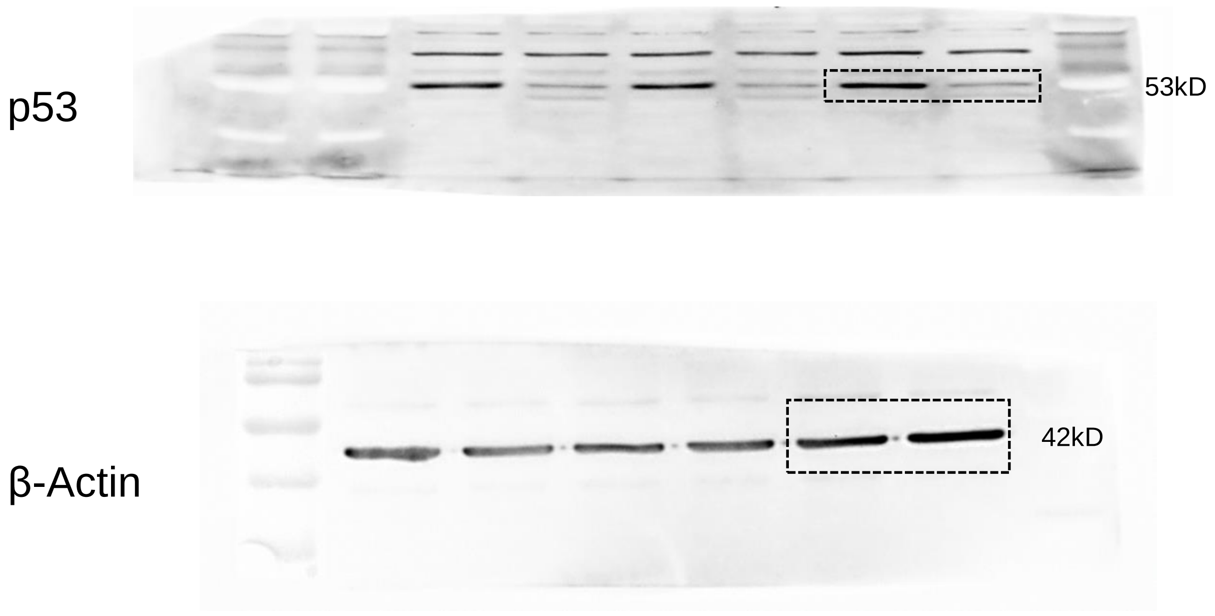

Figure S1 Full-length images of the immunoblots in Figure 1a. Black dot line boxes indicate the cropped images used in Figure 1a.

Figure S2

p62

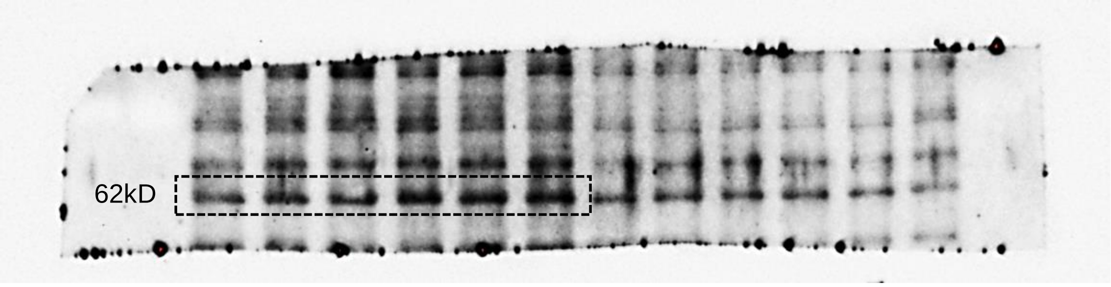

LC3

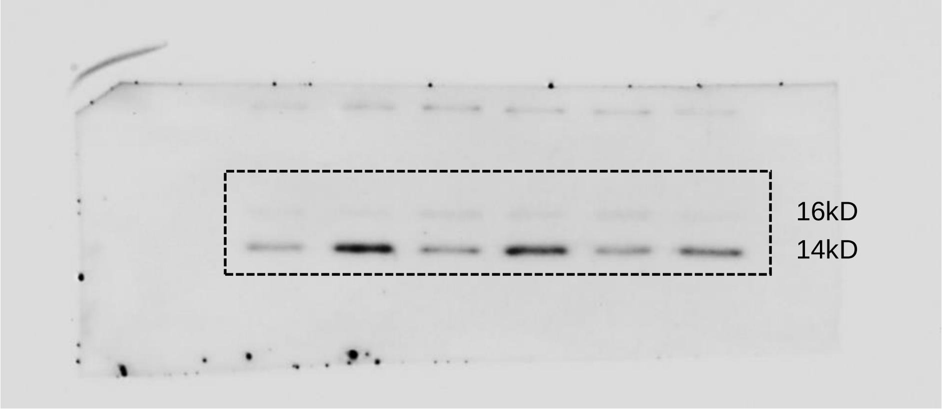

β-Actin

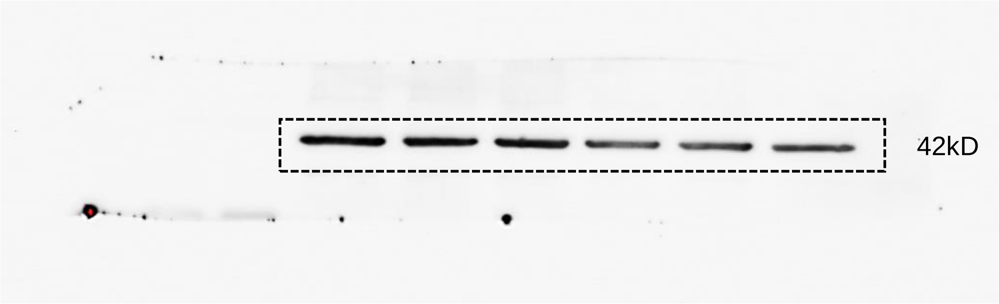

Figure S2 Full-length images of the immunoblots in Figure 1d. Black dot line boxes indicate the cropped images used in Figure 1d.

Figure S3

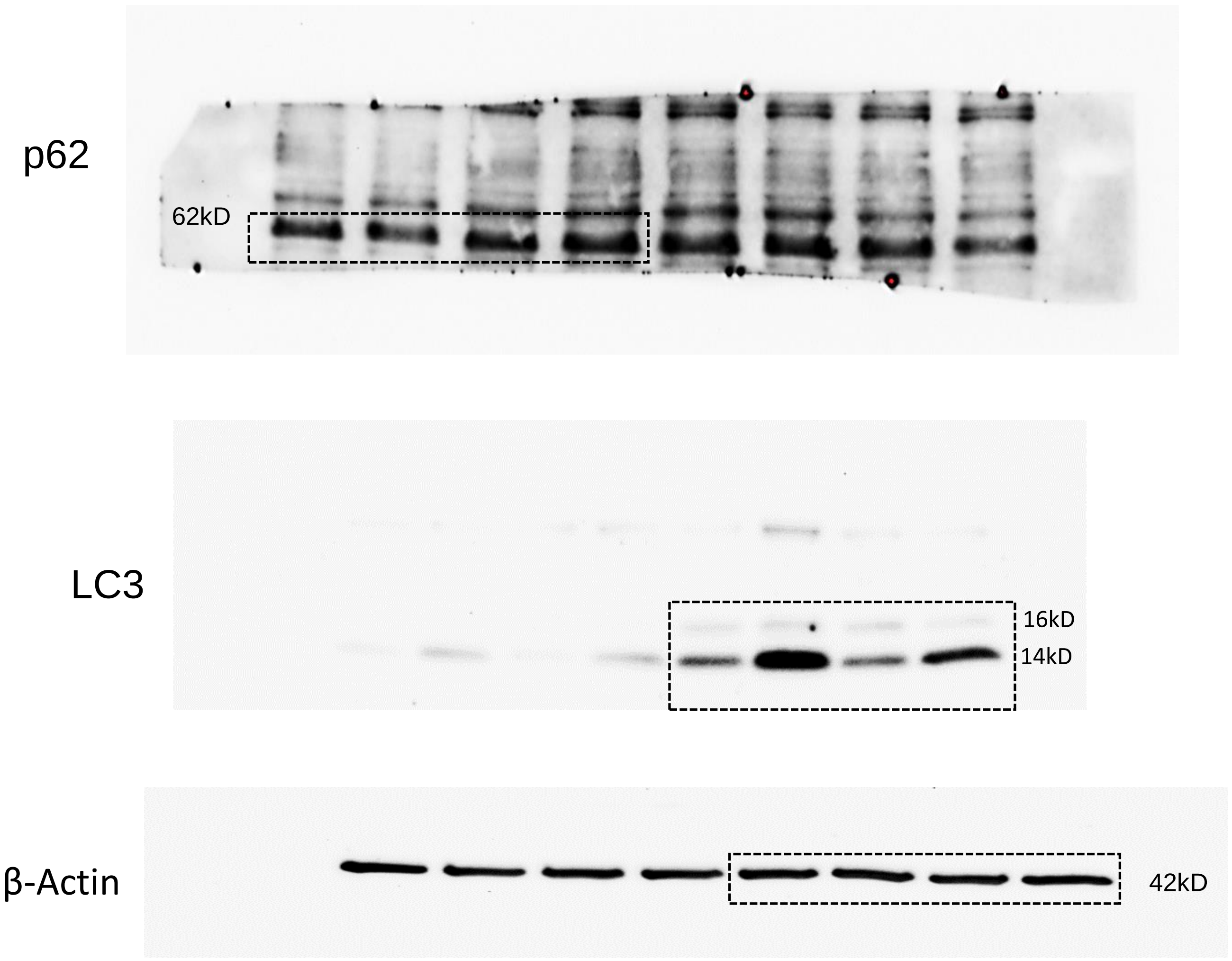

Figure S3 Full-length images of the immunoblots in Figure 1e. Black dot line boxes indicate the cropped images used in Figure 1e.

Figure S4

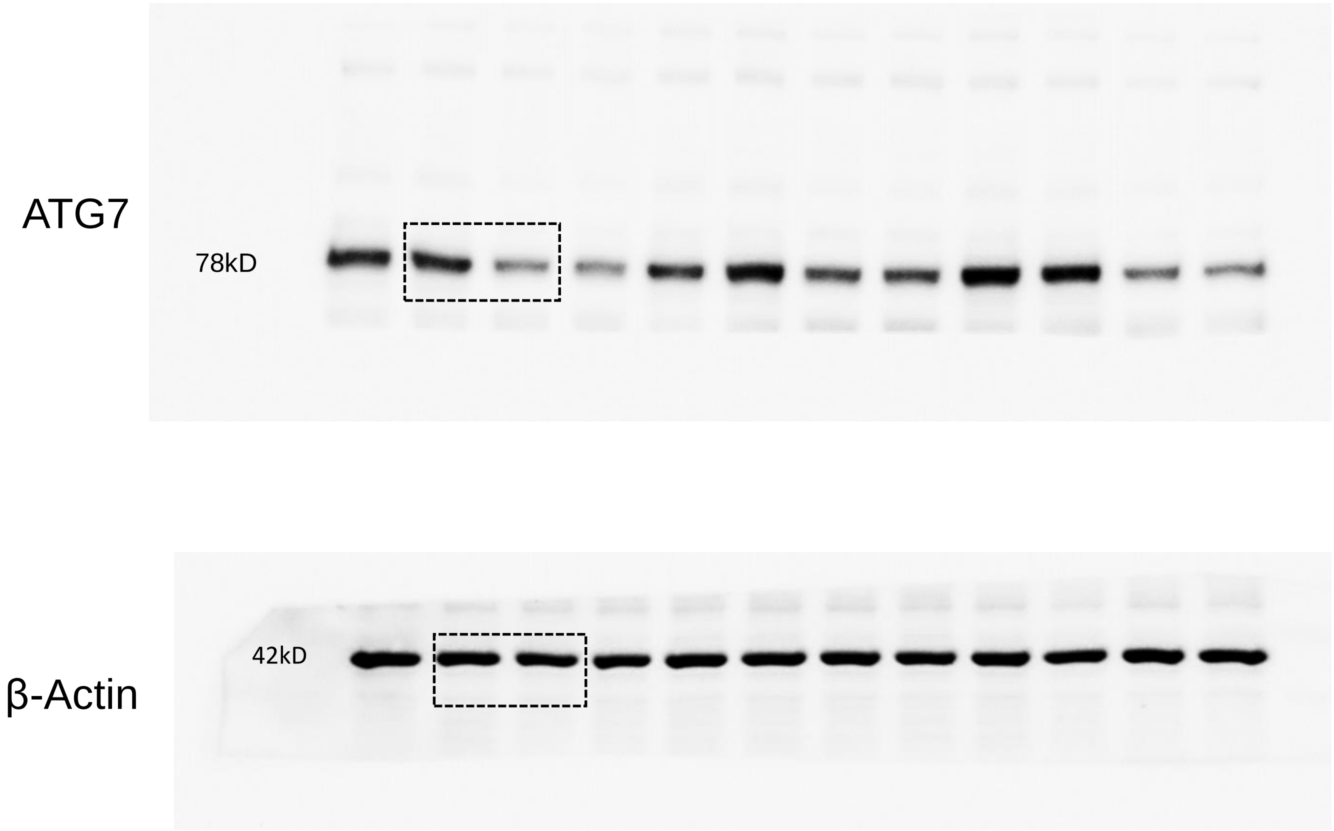

Figure S4 Full-length images of the immunoblots in Figure 2a. Black dot line boxes indicate the cropped images used in Figure 2a.

Figure S5

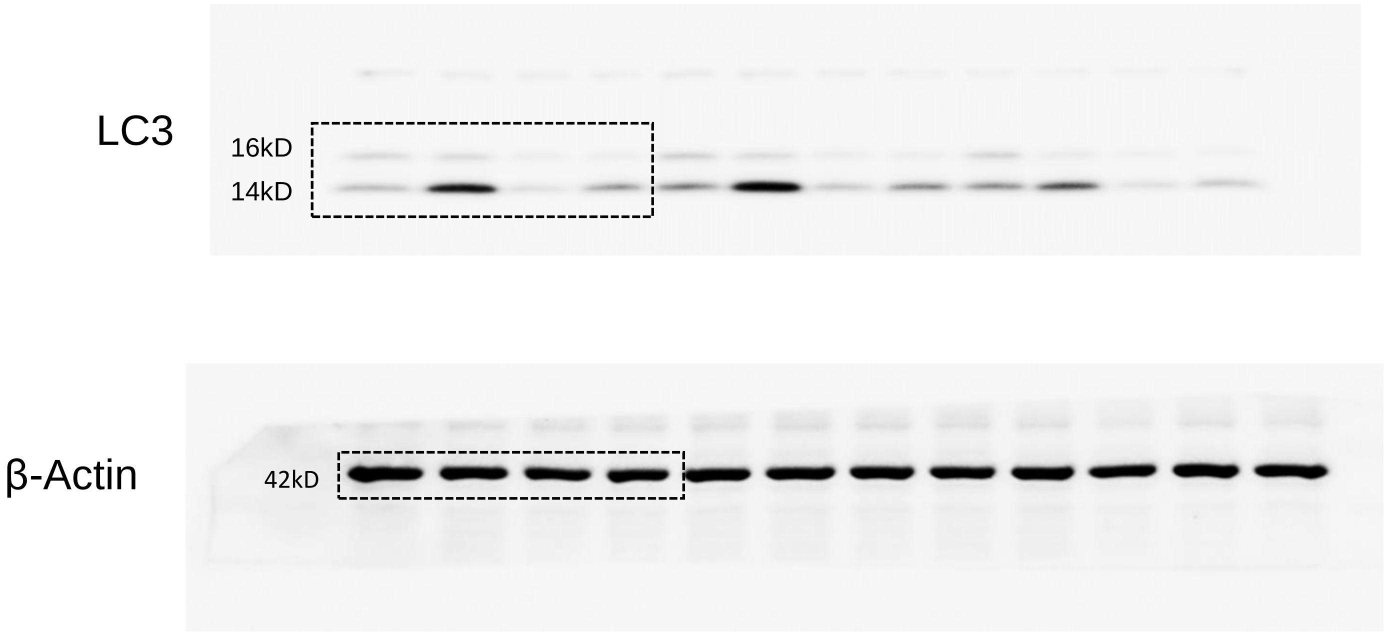

Figure S5 Full-length images of the immunoblots in Figure 2b. Black dot line boxes indicate the cropped images used in Figure 2b.

Figure S6

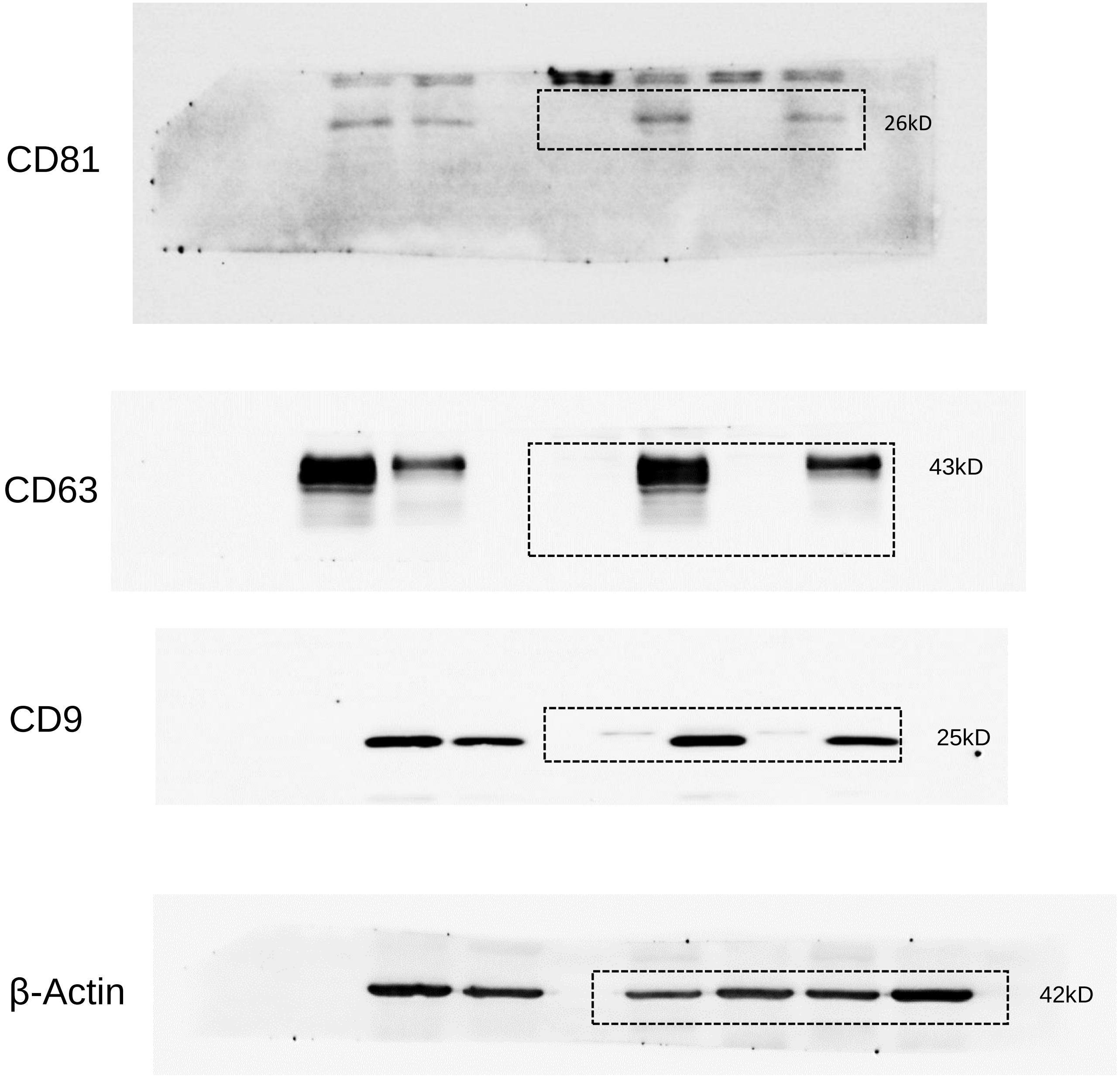

Figure S6 Full-length images of the immunoblots in Figure 3b. Black dot line boxes indicate the cropped images used in Figure 3b.

Figure S7

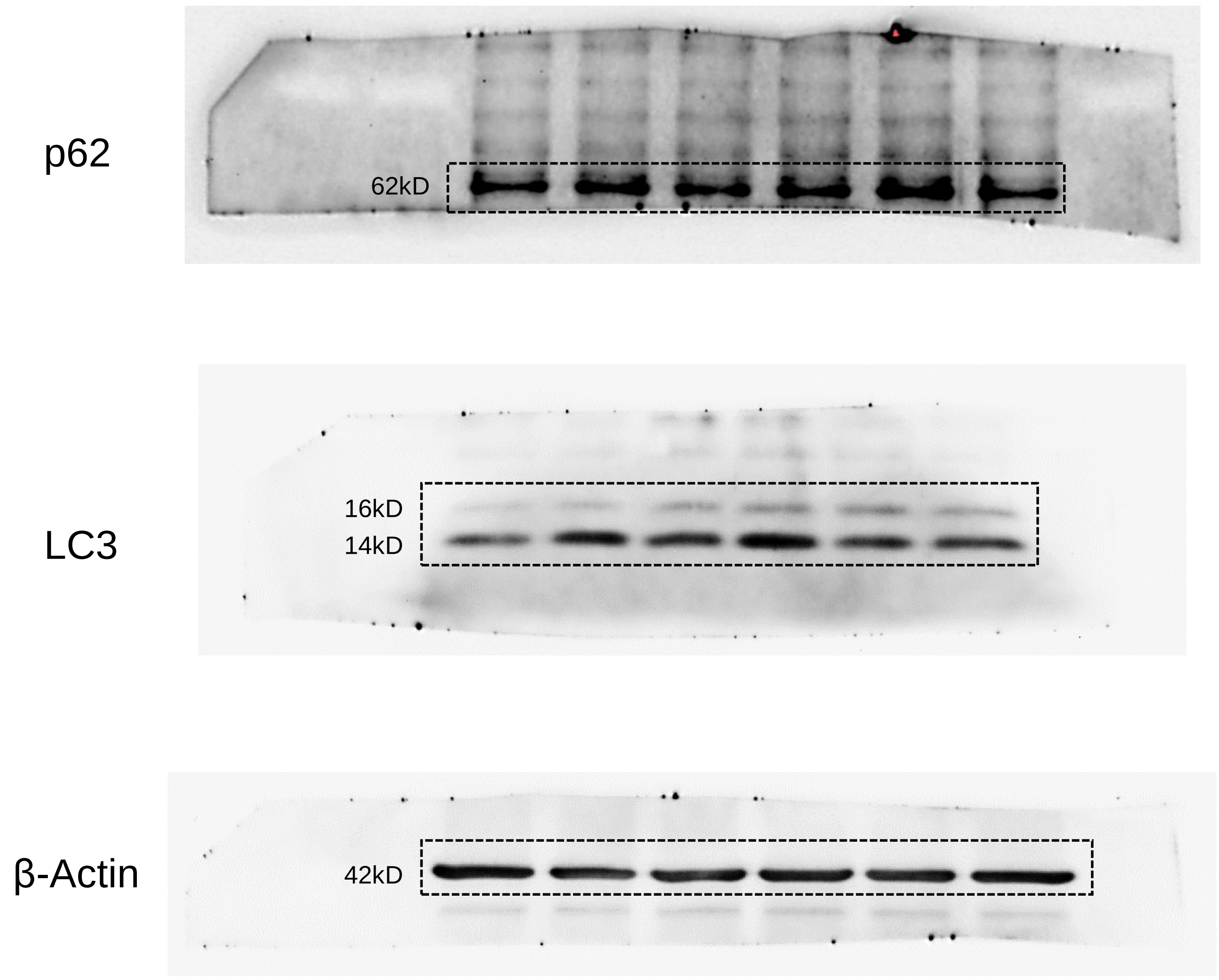

Figure S7 Full-length images of the immunoblots in Figure 3c. Black dot line boxes indicate the cropped images used in Figure 3c.

Figure S8

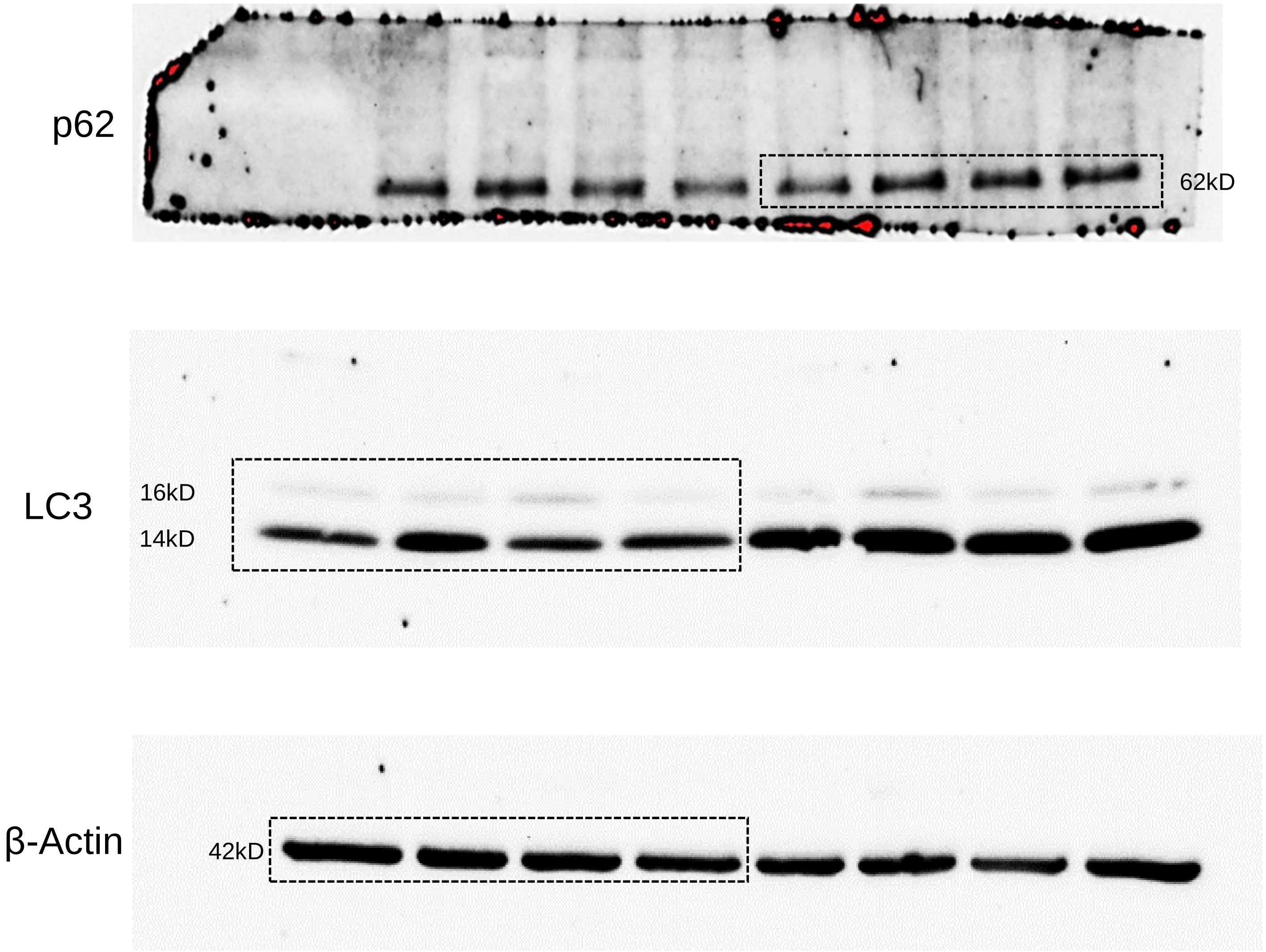

Figure S8 Full-length images of the immunoblots in Figure 3d. Black dot line boxes indicate the cropped images used in Figure 3d.

Figure S9

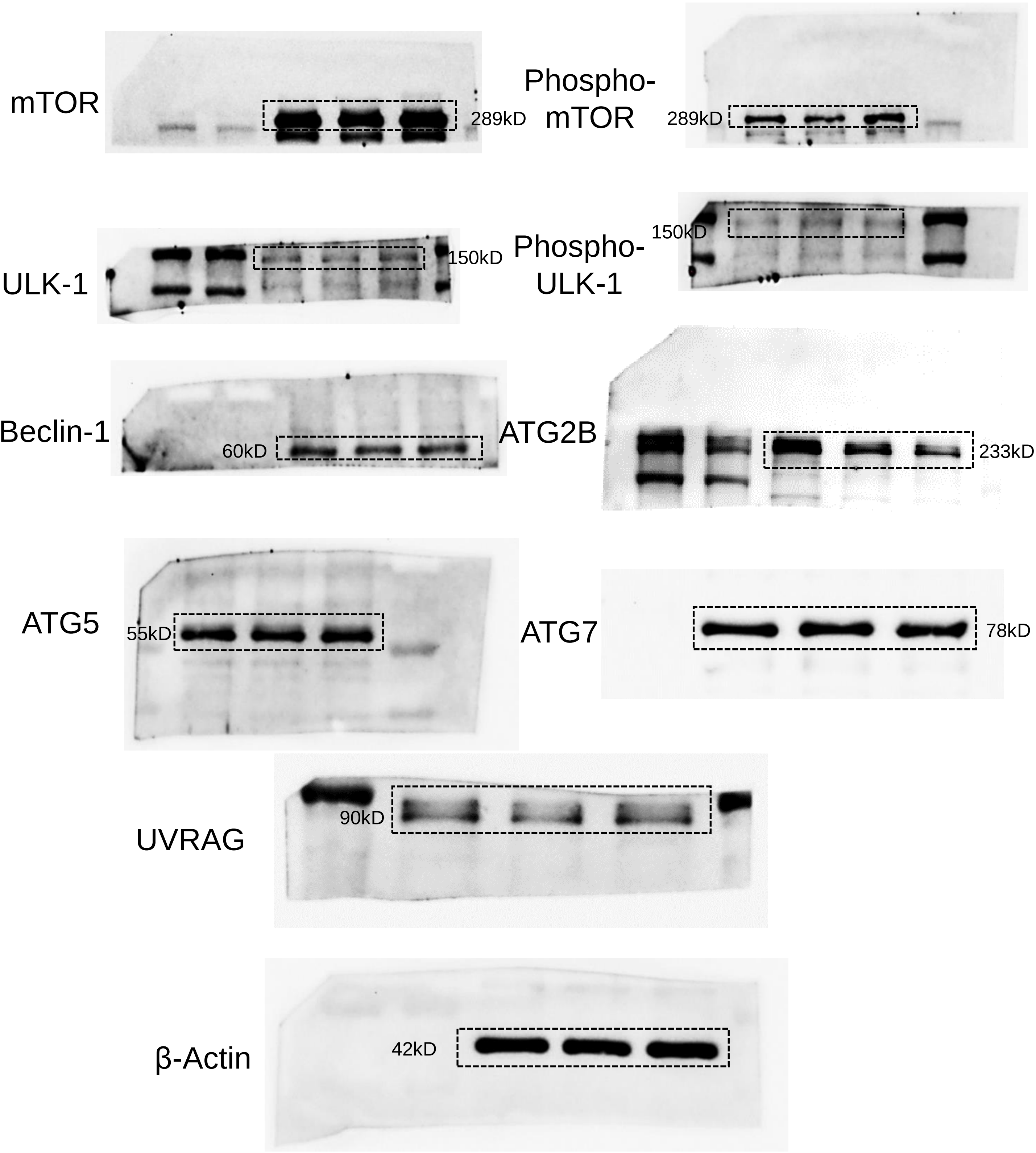

Figure S9 Full-length images of the immunoblots in Figure 4a. Black dot line boxes indicate the cropped images used in Figure 4a.

Figure S10

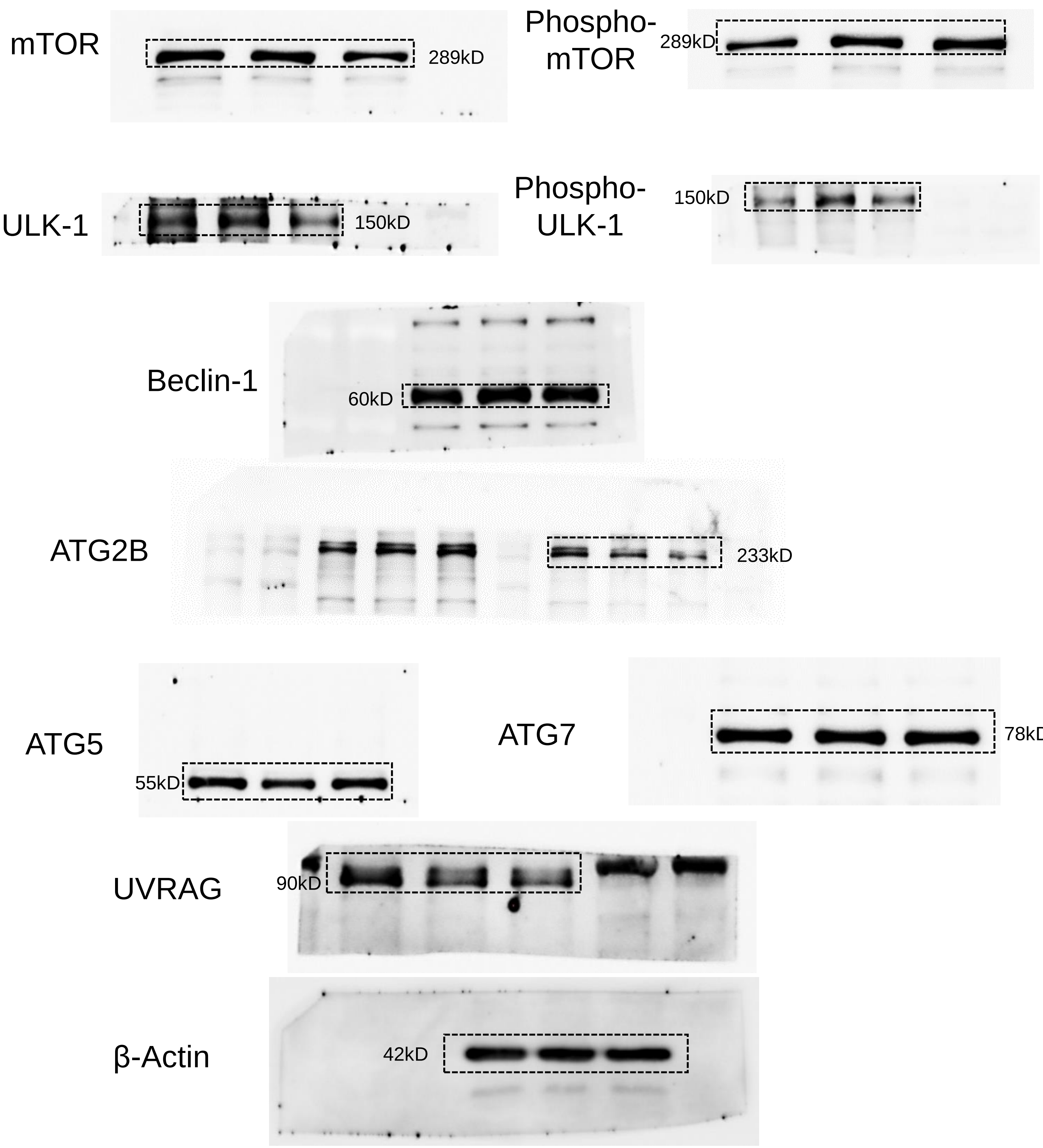

Figure S10 Full-length images of the immunoblots in Figure 4b. Black dot line boxes indicate the cropped images used in Figure 4b.

Figure S11

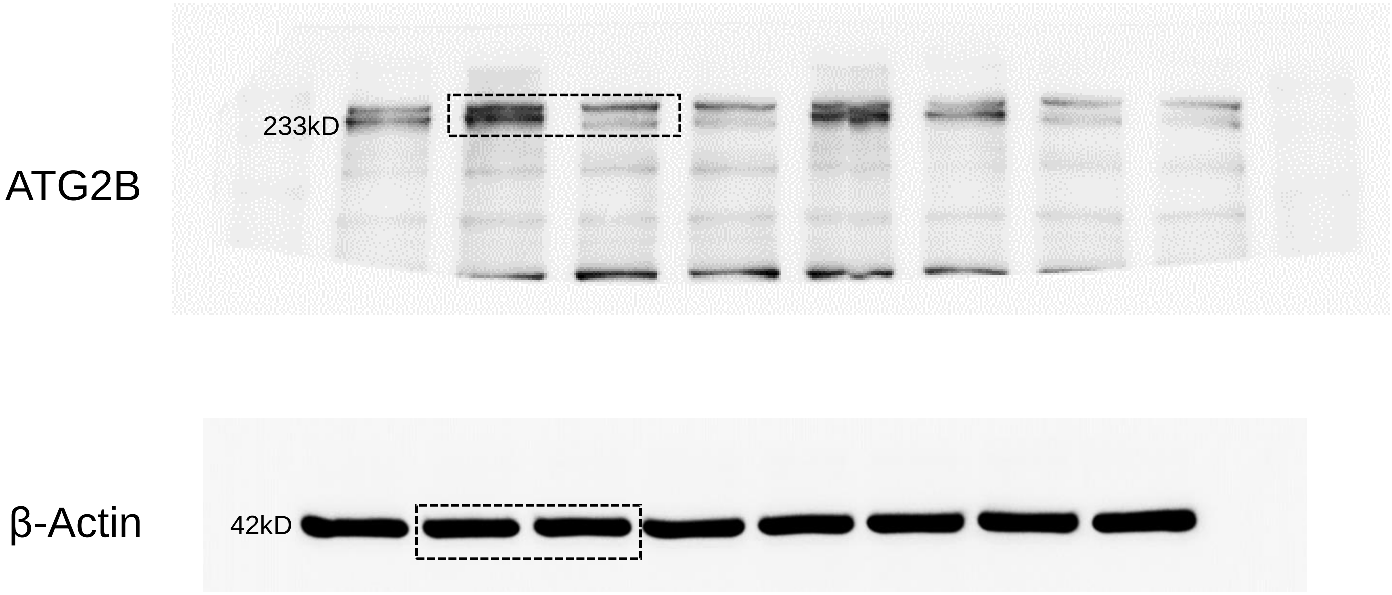

Figure S11 Full-length images of the immunoblots in Figure 4c. Black dot line boxes indicate the cropped images used in Figure 4c.

Figure S12

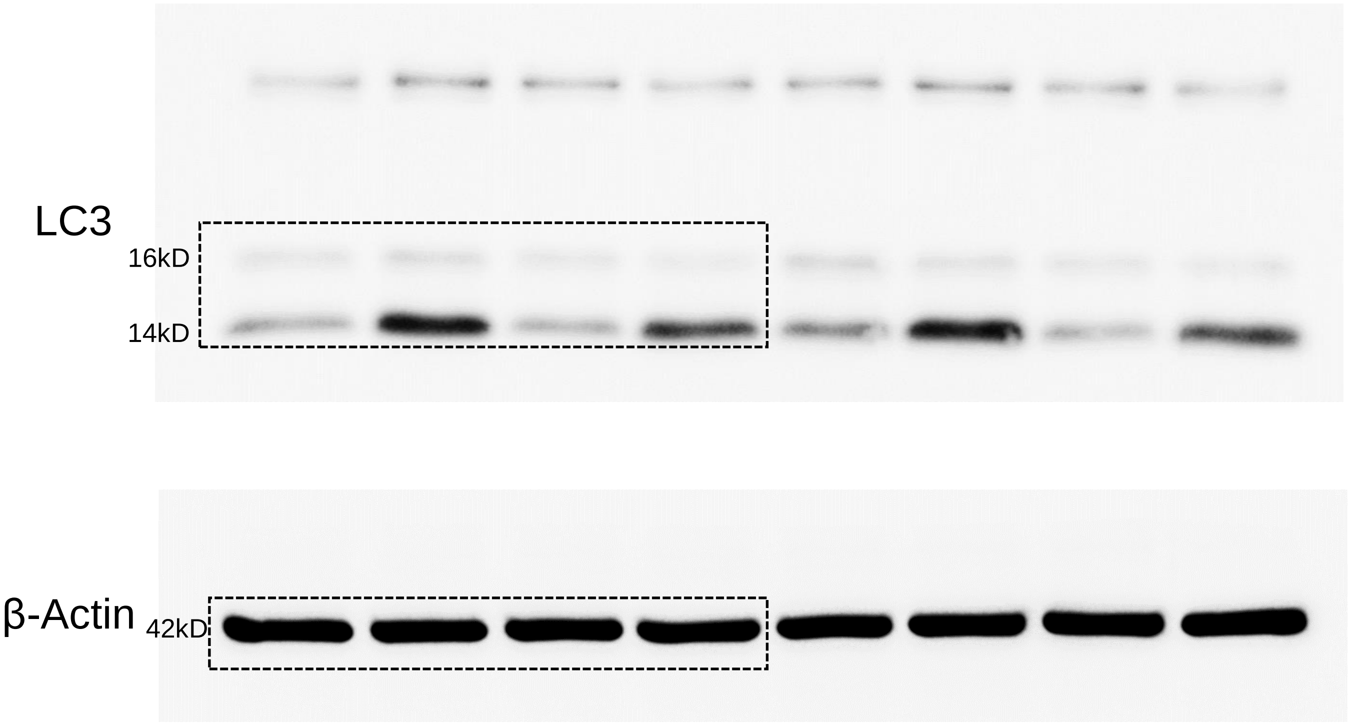

Figure S12 Full-length images of the immunoblots in Figure 4d. Black dot line boxes indicate the cropped images used in Figure 4d.

Figure S13

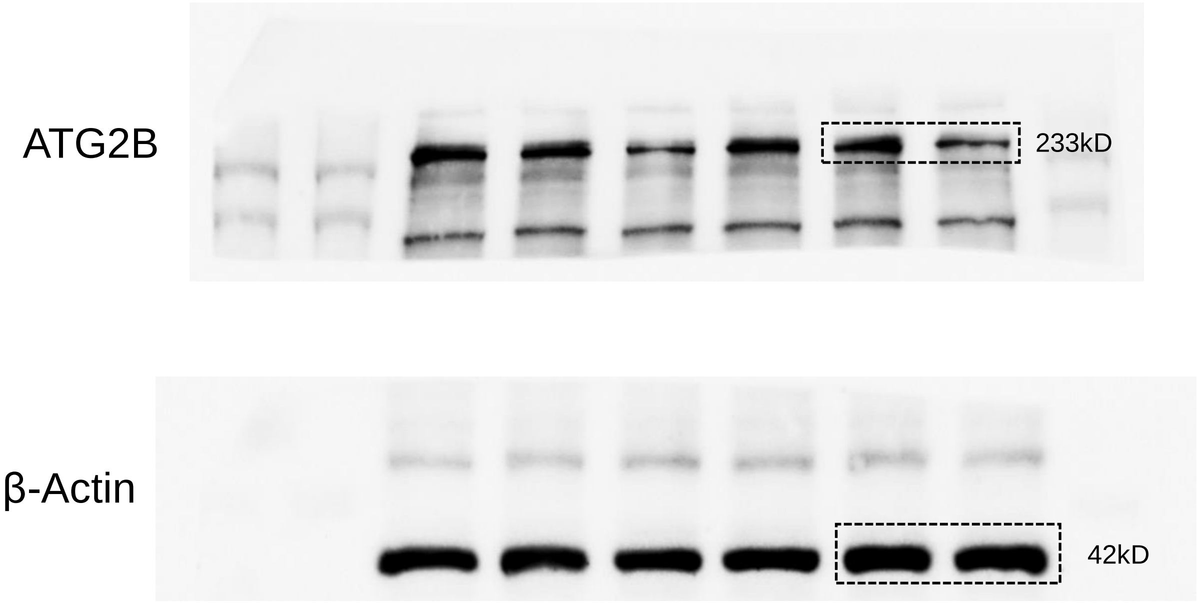

Figure S13 Full-length images of the immunoblots in Figure 5d. Black dot line boxes indicate the cropped images used in Figure 5d.

Figure S14

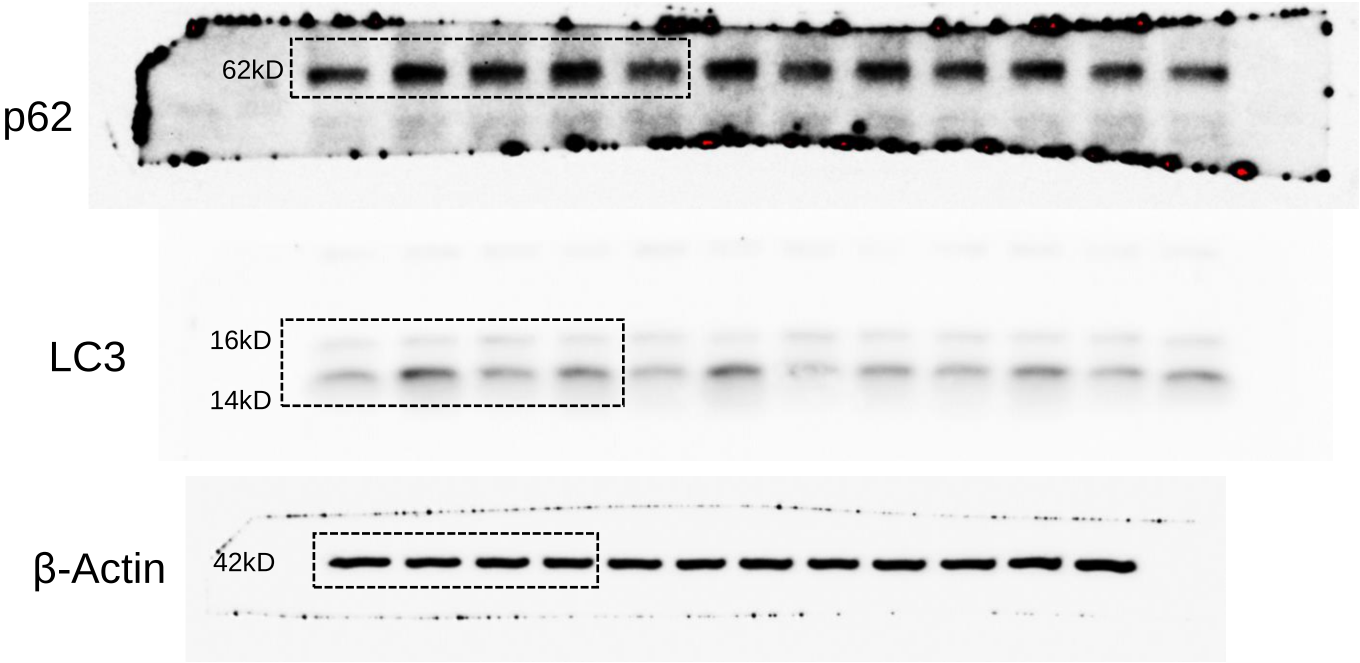

Figure S14 Full-length images of the immunoblots in Figure 5e. Black dot line boxes indicate the cropped images used in Figure 5e.

hsa-miR-3667-5p, hsa-miR-630, hsa-miR-4261, hsa-miR-6886-5p, hsa-miR-5703, hsa-miR-6837-5p, hsa-miR-181a-5p, hsa-miR-6810-5p, hsa-miR-4635, hsa-miR-4736, hsa-miR-887-5p, hsa-miR-6835-5p, hsa-miR-6821-3p, hsa-miR-4738-3p, hsa-miR-5581-3p, hsa-miR-6833-3p, hsa-miR-432-3p, hsa-miR-381-5p, hsa-let-7f-1-3p, hsa-miR-6830-3p, hsa-miR-3127-5p, hsa-miR-664a-3p, hsa-miR-6847-3p, hsa-miR-320c, hsa-miR-6758-5p, hsa-miR-6780b-5p, hsa-miR-4785, hsa-miR-6761-5p, hsa-miR-199a-5p, hsa-miR-583, hsa-miR-6799-5p, hsa-miR-433-5p, hsa-miR-575, hsa-miR-29c-3p, hsa-miR-6879-5p, hsa-miR-7856-5p, hsa-miR-4652-3p, hsa-miR-6745, hsa-miR-432-5p, hsa-miR-374c-3p, hsa-miR-1202, hsa-miR-4442, hsa-miR-3190-3p, hsa-miR-320d, hsa-miR-216b-3p, hsa-miR-6833-5p, hsa-miR-200a-3p, hsa-miR-4740-3p, hsa-miR-3651, hsa-miR-483-5p, hsa-miR-181d-3p, hsa-miR-4326, hsa-miR-6824-5p, hsa-miR-6831-5p, hsa-miR-211-5p, hsa-miR-6124, hsa-miR-6773-3p, hsa-miR-4638-5p, hsa-miR-3155b, hsa-miR-6716-3p, hsa-miR-378f, hsa-miR-6855-3p, hsa-miR-6127, hsa-miR-6716-5p, hsa-miR-4745-3p, hsa-miR-6870-5p, hsa-miR-7111-5p, hsa-miR-4419a, hsa-miR-3972, hsa-miR-668-3p, hsa-miR-6133, hsa-miR-4725-3p, hsa-miR-5698, hsa-miR-7151-3p, hsa-miR-4716-3p, hsa-miR-6767-5p, hsa-miR-6086, hsa-miR-4436a, hsa-miR-5580-3p, hsa-miR-6775-5p, hsa-miR-6785-5p, hsa-miR-4776-3p, hsa-miR-6881-3p, hsa-miR-6742-3p, hsa-miR-6876-5p, hsa-miR-3198, hsa-miR-4476, hsa-miR-708-5p, hsa-miR-3191-5p, hsa-miR-7109-5p, hsa-miR-491-5p, hsa-miR-4267, hsa-miR-4723-5p, hsa-miR-5699-5p, hsa-miR-7111-3p, hsa-miR-204-3p, hsa-miR-330-3p, hsa-miR-146b-3p, hsa-miR-548ab, hsa-miR-1203, hsa-miR-493-3p, hsa-miR-3918, hsa-miR-4285, hsa-miR-4698, hsa-miR-765, hsa-miR-3679-5p, hsa-miR-6760-3p, hsa-miR-378i, hsa-miR-1908-5p, hsa-miR-7106-5p, hsa-miR-1303, hsa-miR-671-5p, hsa-miR-7845-5p, hsa-miR-605-5p, hsa-miR-3064-5p, hsa-miR-513b-5p, hsa-miR-3622b-5p, hsa-miR-6772-3p, hsa-miR-5196-5p, hsa-miR-659-3p, hsa-miR-4270, hsa-miR-519d-3p, hsa-miR-6751-3p, hsa-miR-6878-3p, hsa-miR-483-3p, hsa-miR-4800-5p, hsa-miR-1914-5p, hsa-miR-629-3p, hsa-miR-6085, hsa-miR-4727-3p, hsa-miR-6751-5p, hsa-miR-4682, hsa-miR-4297, hsa-miR-6809-3p, hsa-miR-3944-5p, hsa-miR-1207-5p, hsa-miR-572, hsa-miR-6892-5p, hsa-miR-4748, hsa-miR-3153, hsa-miR-4534, hsa-miR-6819-5p, hsa-miR-7704, hsa-miR-30c-1-3p, hsa-miR-1185-1-3p, hsa-miR-668-5p, hsa-miR-4515, hsa-miR-6871-3p, hsa-miR-642b-3p, hsa-miR-7853-5p, hsa-miR-6512-3p, hsa-miR-1296-3p, hsa-miR-6861-3p, hsa-miR-6813-3p, hsa-miR-3141, hsa-miR-150-5p, hsa-miR-34b-3p, hsa-miR-1236-3p, hsa-miR-4755-3p, hsa-miR-2276-3p, hsa-miR-3655, hsa-miR-3945, hsa-miR-4722-3p, hsa-miR-8078, hsa-miR-4305, hsa-miR-4283, hsa-miR-2277-3p, hsa-miR-4321, hsa-miR-3202, hsa-miR-3667-3p, hsa-miR-6891-5p, hsa-miR-6881-5p, hsa-miR-1275, hsa-miR-6822-3p, hsa-miR-877-5p, hsa-miR-6856-5p, hsa-miR-4725-5p, hsa-miR-492, hsa-miR-4531, hsa-miR-186-3p, hsa-miR-4714-5p, hsa-miR-1180-5p, hsa-miR-485-3p, hsa-miR-661, hsa-miR-6883-3p, hsa-miR-6749-5p, hsa-miR-150-3p, hsa-miR-4732-3p, hsa-miR-6769a-5p, hsa-miR-3131, hsa-miR-6759-3p, hsa-miR-6867-3p, hsa-miR-7109-3p

Figure S15. List of 193 microRNAs
Relative expression ratio≧2
(sh p53 Exosome/ sh control Exosome)
