# Supplementary material for: Suppression of autophagy promotes fibroblast activation in p53-deficient colorectal cancer cells
Source: Sci Rep. 2021 Sep 30;11:19524. doi: 10.1038/s41598-021-98865-1 (PMC8484348; doi:10.1038/s41598-021-98865-1)
Supplement: Supplementary file 2 — Supplementary Information 2. [file 41598_2021_98865_MOESM2_ESM.pdf]

Figure S3

LC3

β-Actin

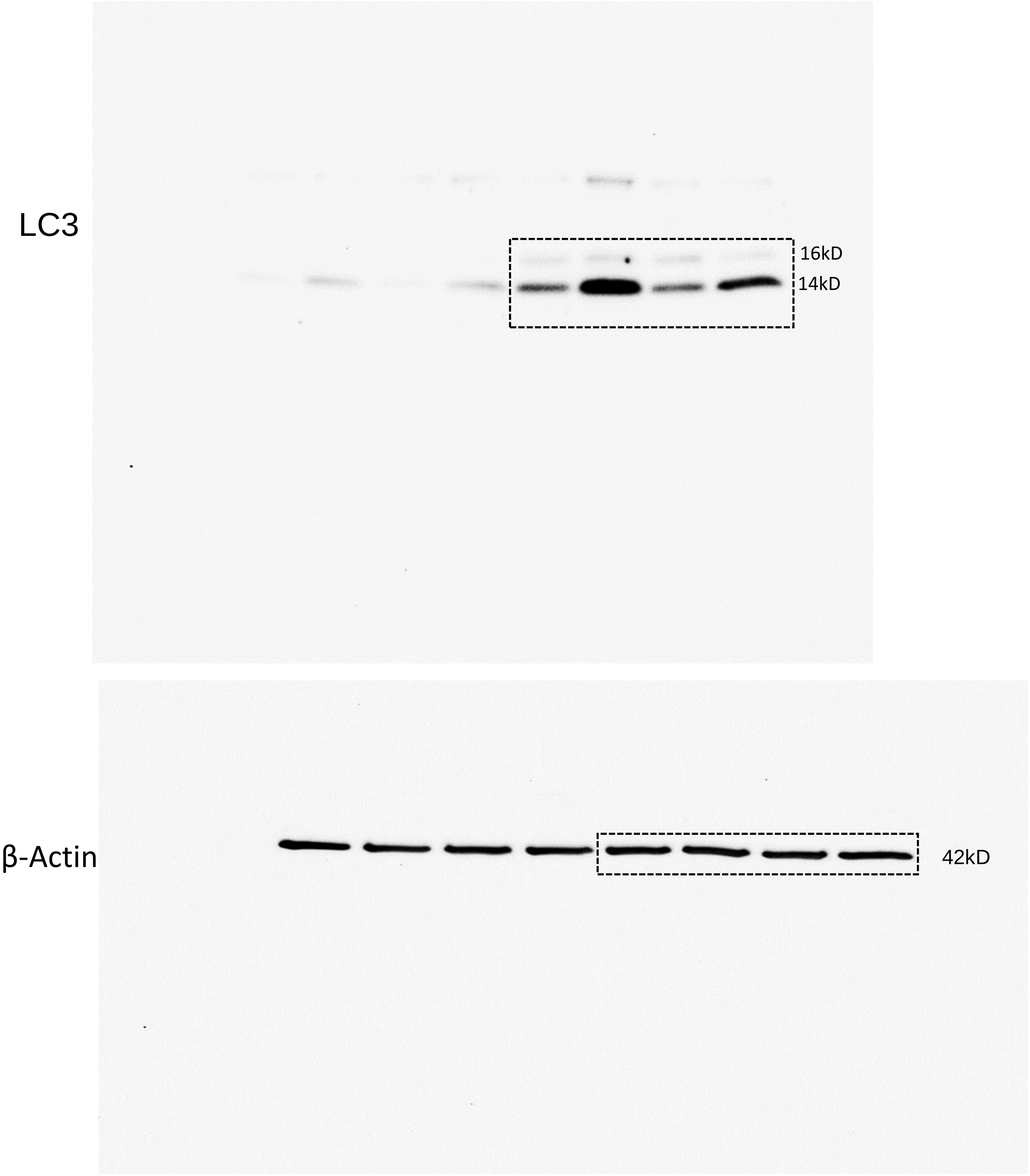

Figure S3 Full-length images of the immunoblots in Figure 1e. Black dot line boxes indicate the cropped images used in Figure 1e.

Figure S6

CD9

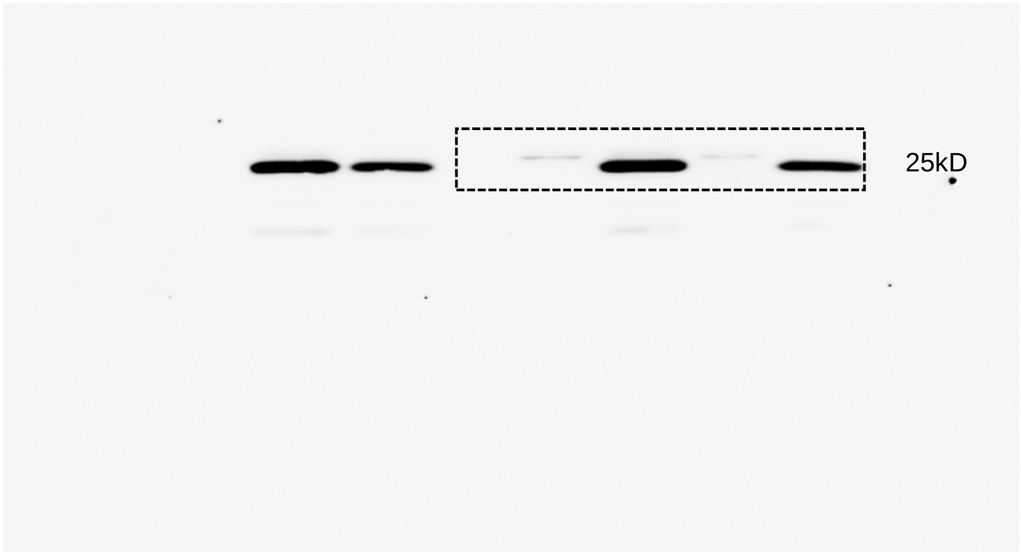

Figure S6 Full-length images of the immunoblots in Figure 3b. Black dot line boxes indicate the cropped images used in Figure 3b.

Figure S8

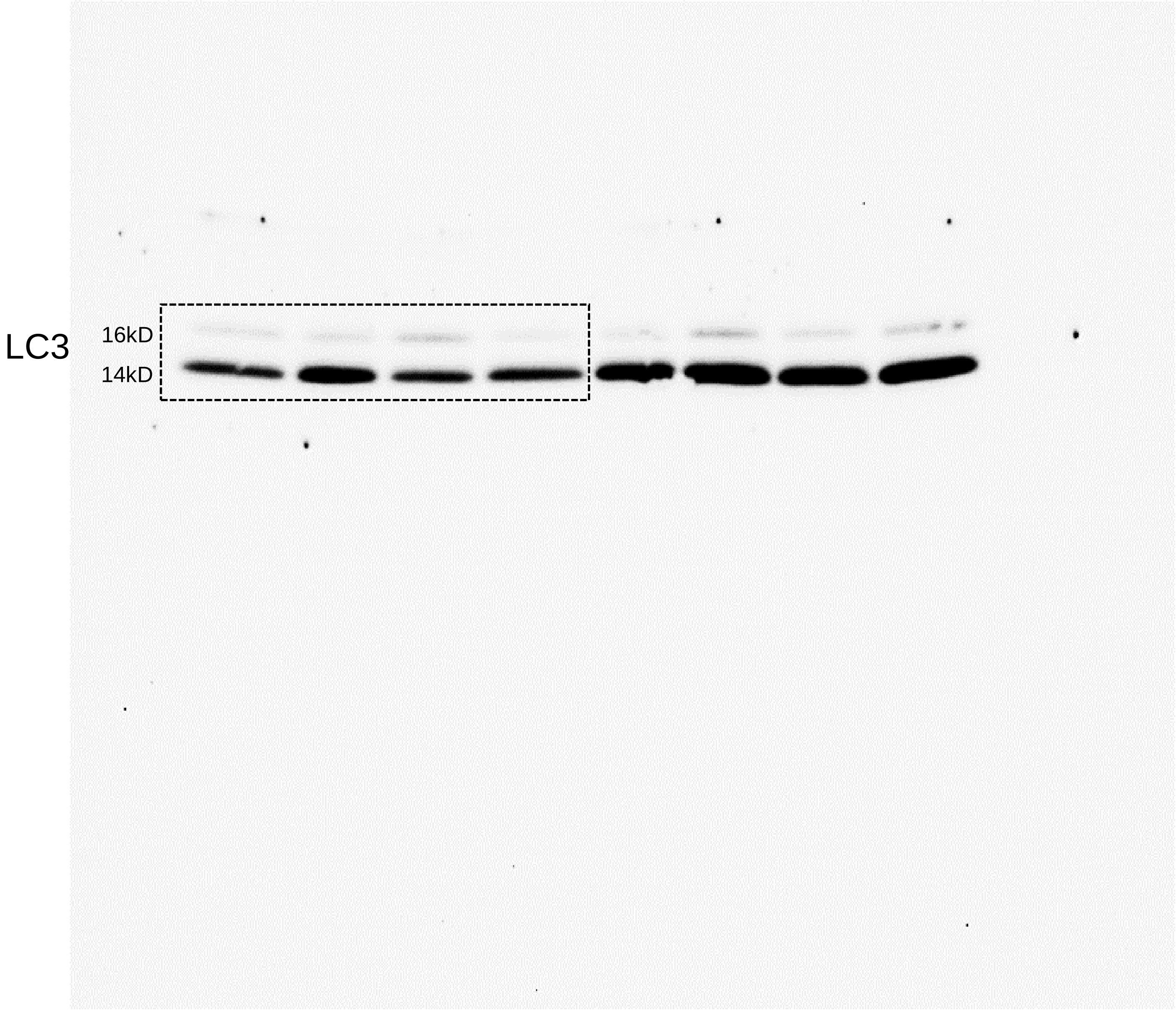

Figure S8 Full-length images of the immunoblots in Figure 3d. Black dot line boxes indicate the cropped images used in Figure 3d.

Figure S8

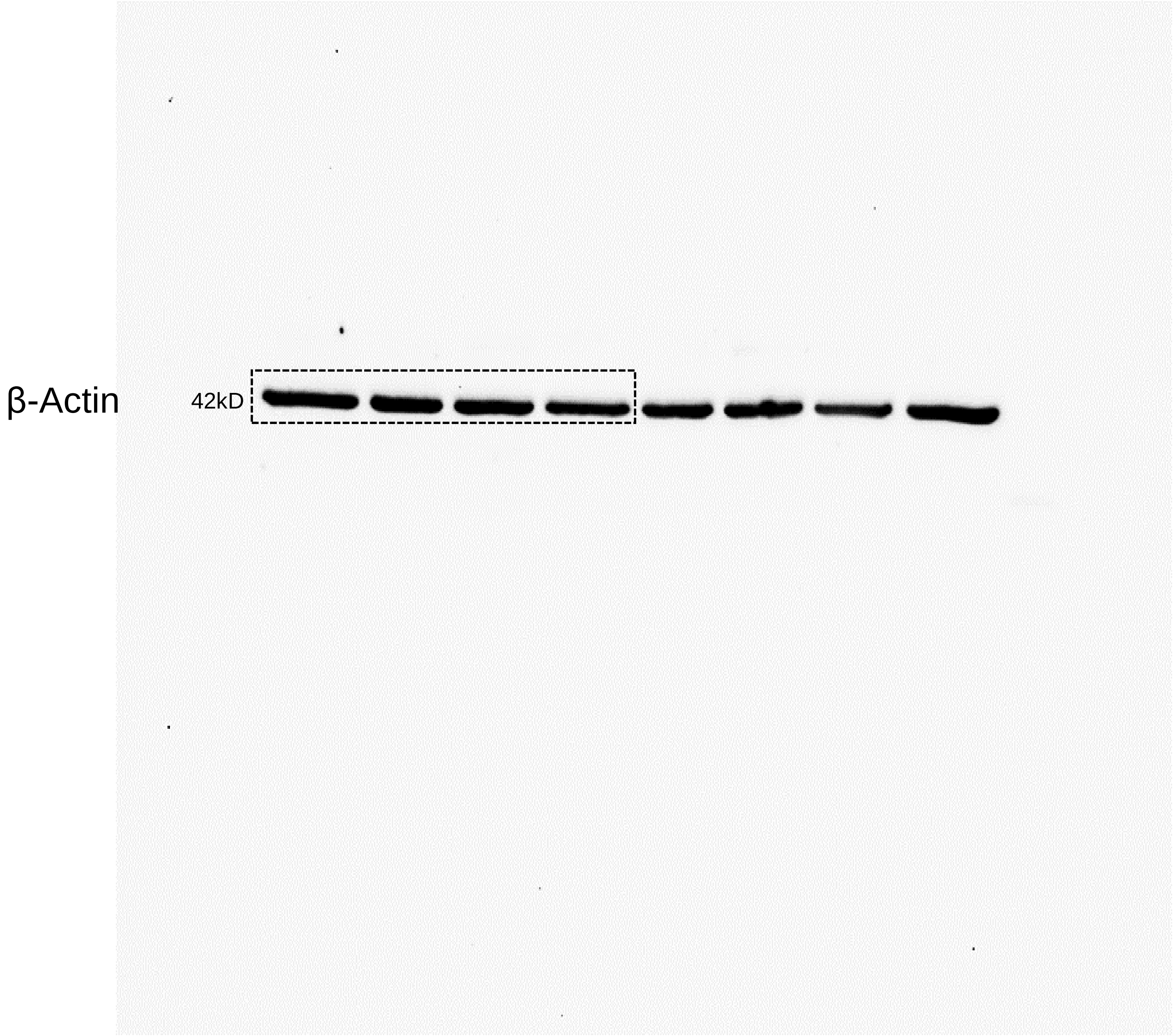

Figure S8 Full-length images of the immunoblots in Figure 3d. Black dot line boxes indicate the cropped images used in Figure 3d.

Figure S10

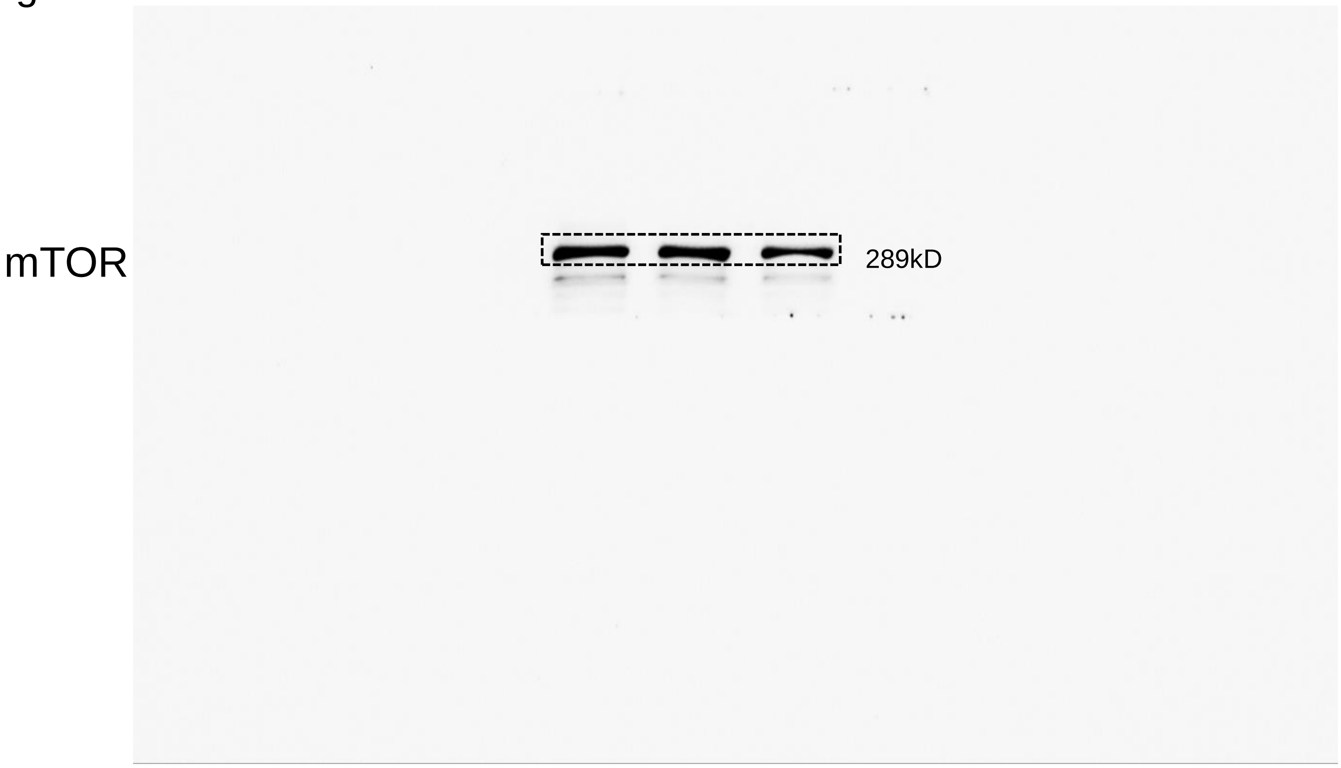

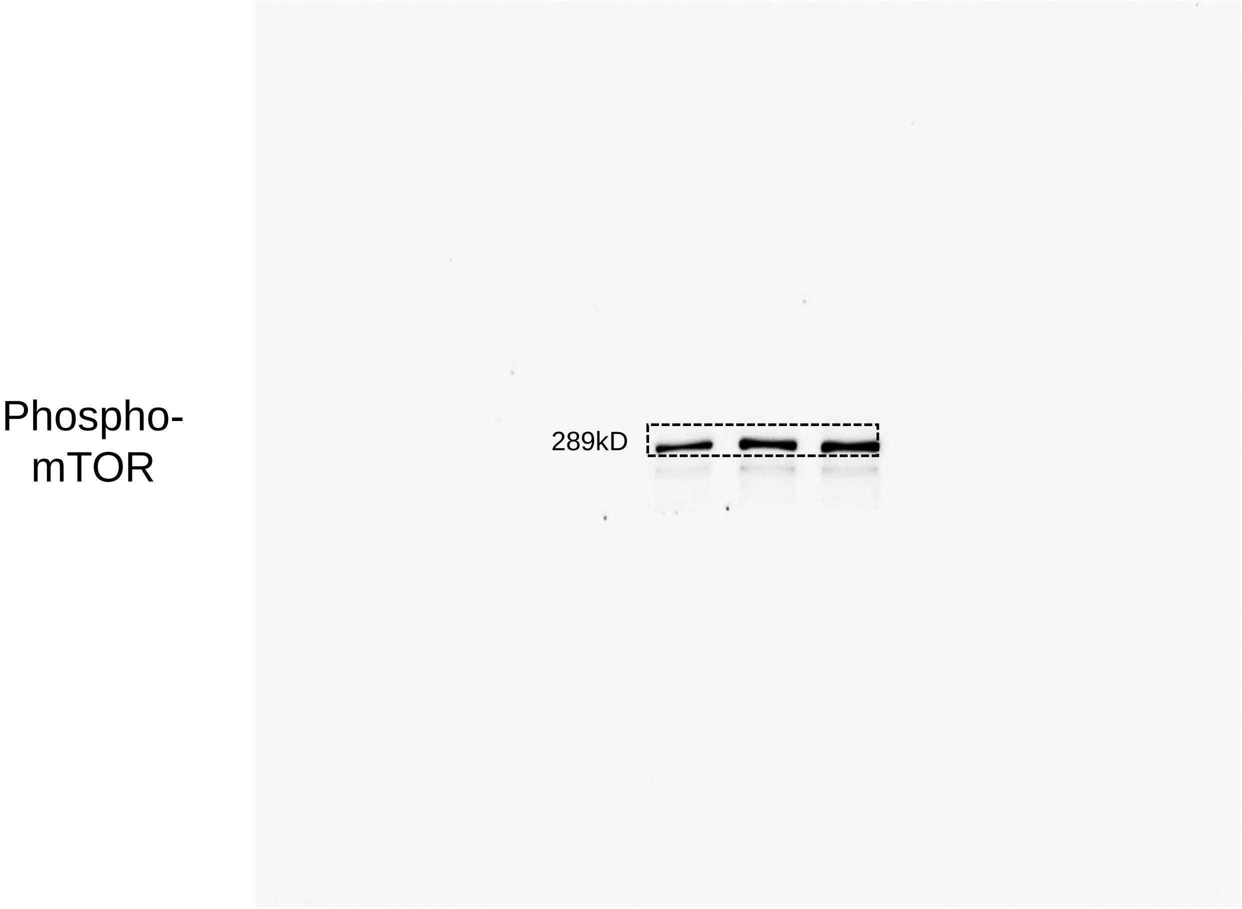

Figure S10 Full-length images of the immunoblots in Figure 4b. Black dot line boxes indicate the cropped images used in Figure 4b.

Figure S10

ULK-1

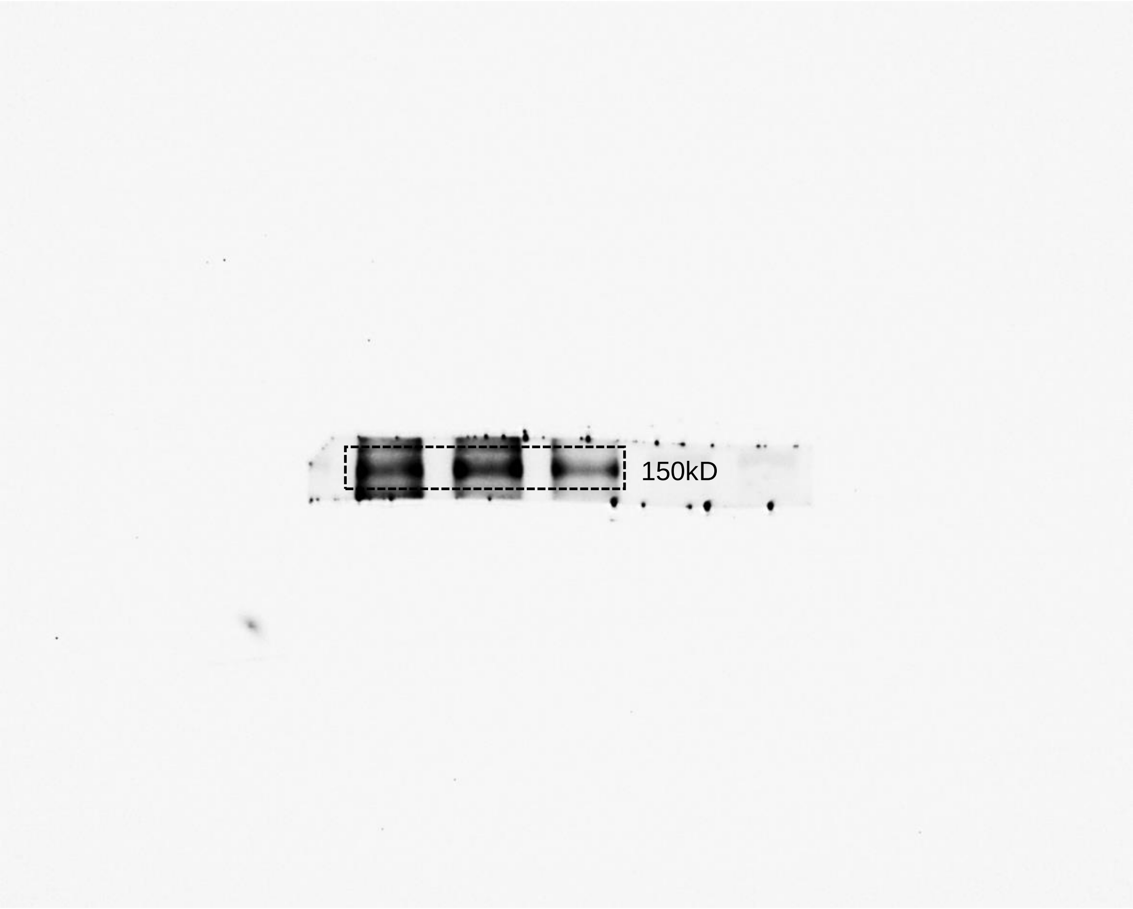

Phospho-
ULK-1

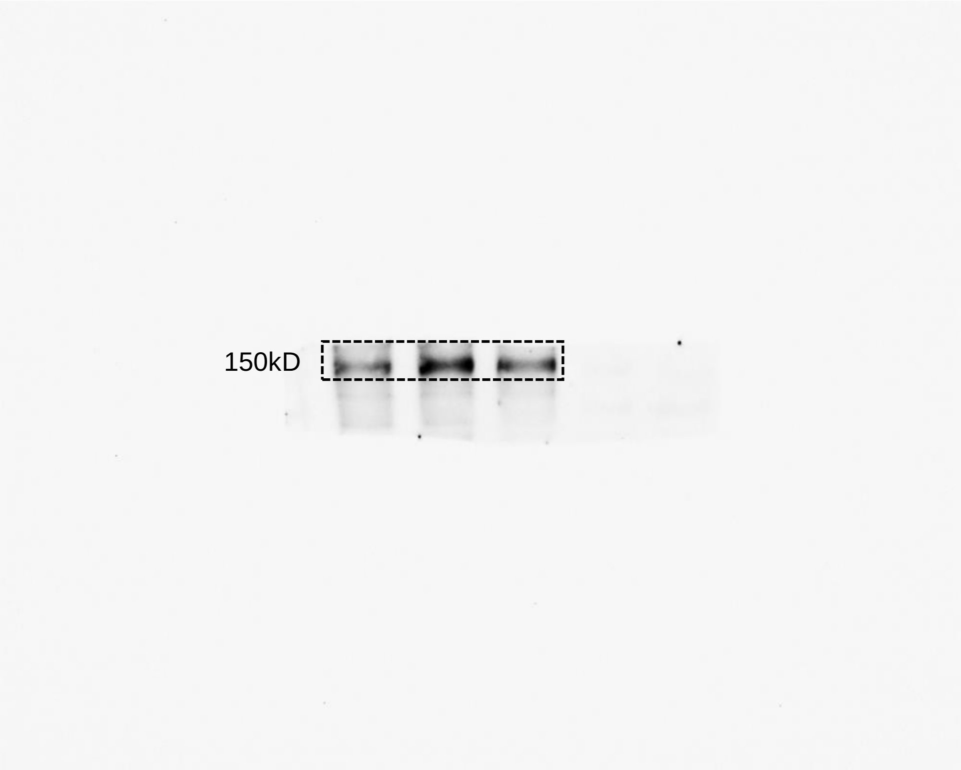

Figure S10 Full-length images of the immunoblots in Figure 4b. Black dot line boxes indicate the cropped images used in Figure 4b.

Figure S10

Beclin-1

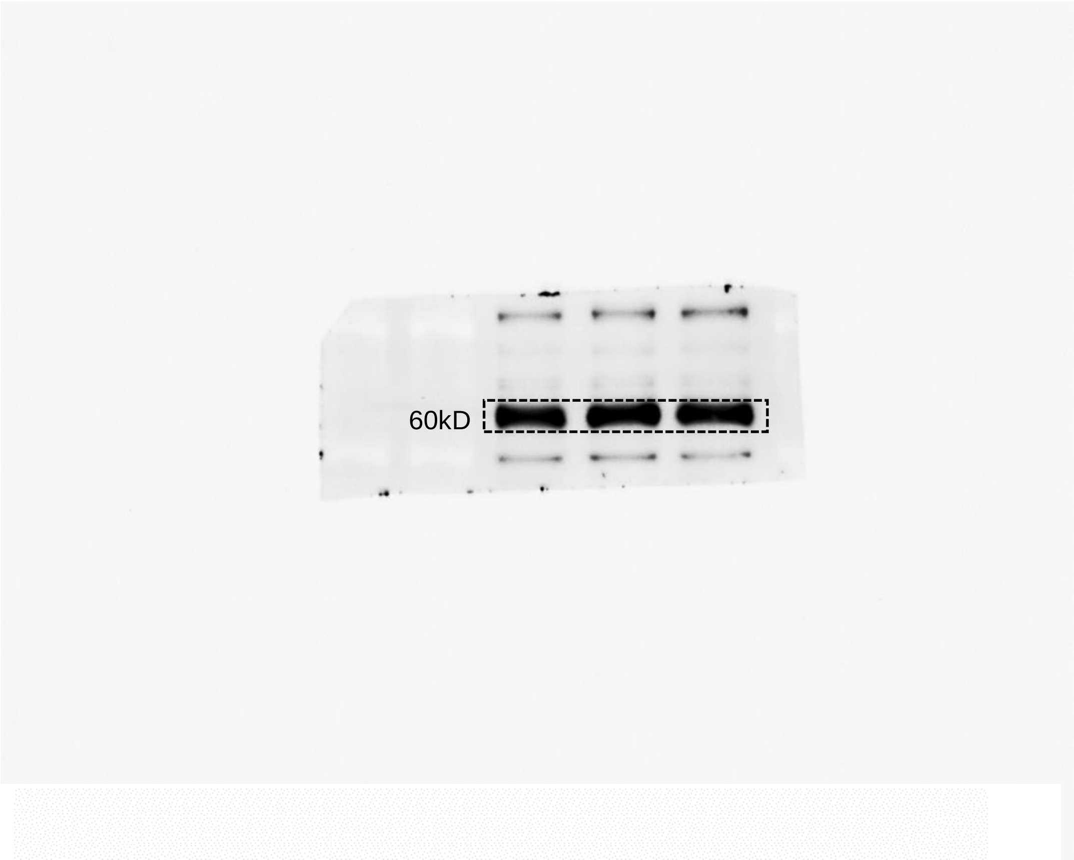

ATG2B

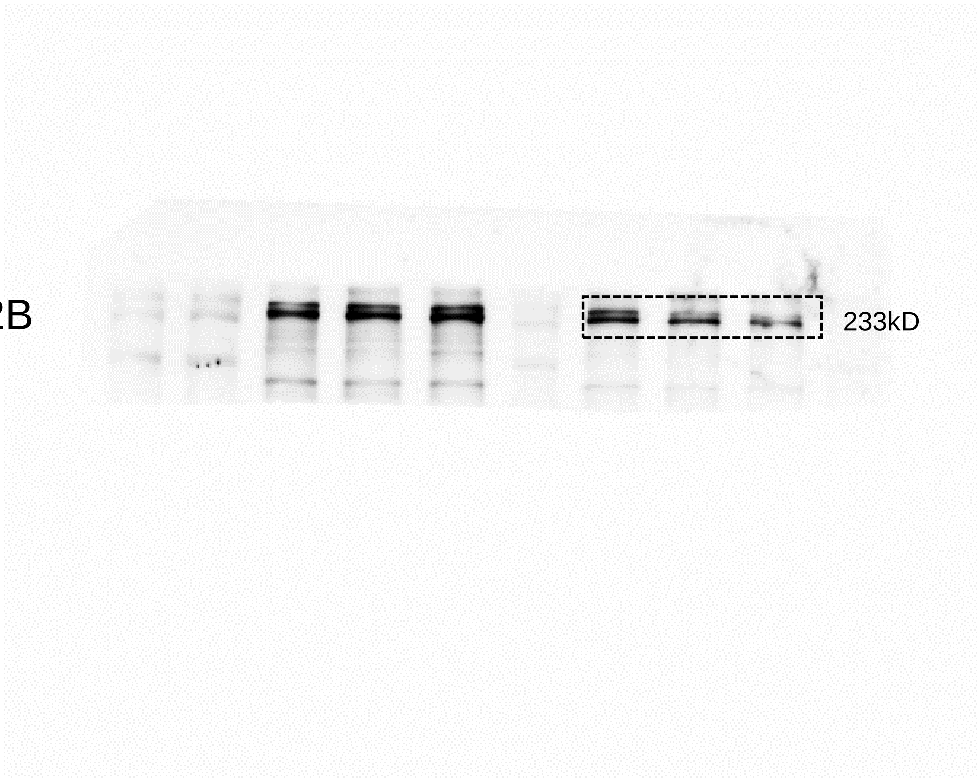

Figure S10 Full-length images of the immunoblots in Figure 4b. Black dot line boxes indicate the cropped images used in Figure 4b.

Figure S10

ATG5

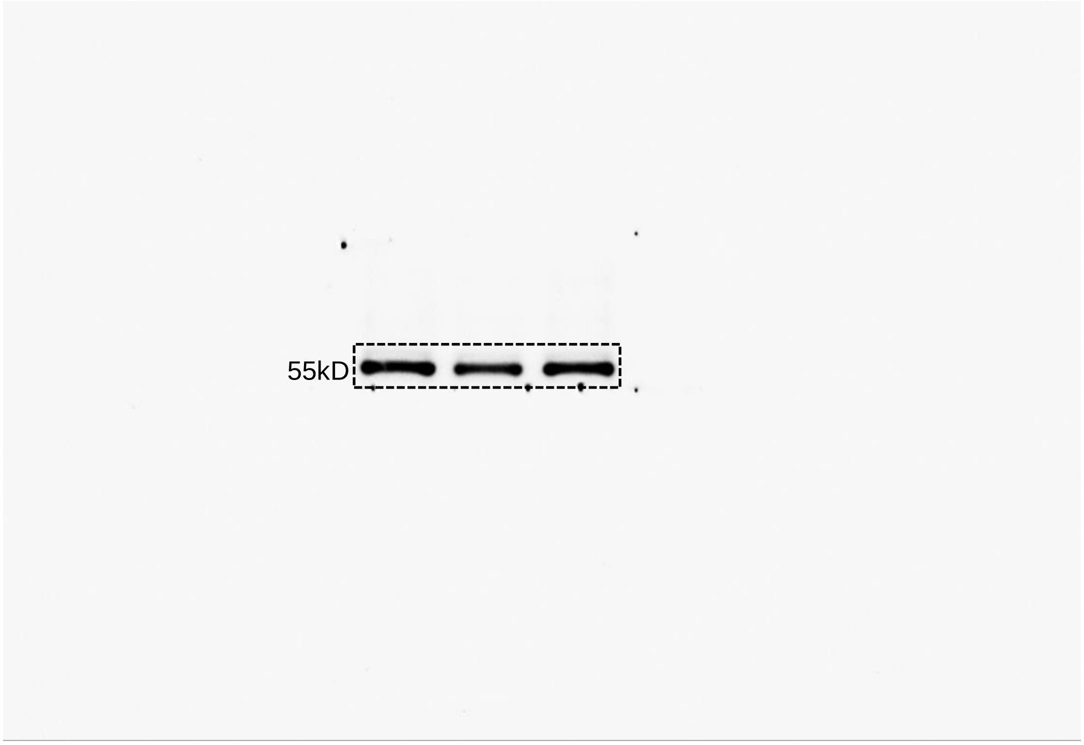

ATG7

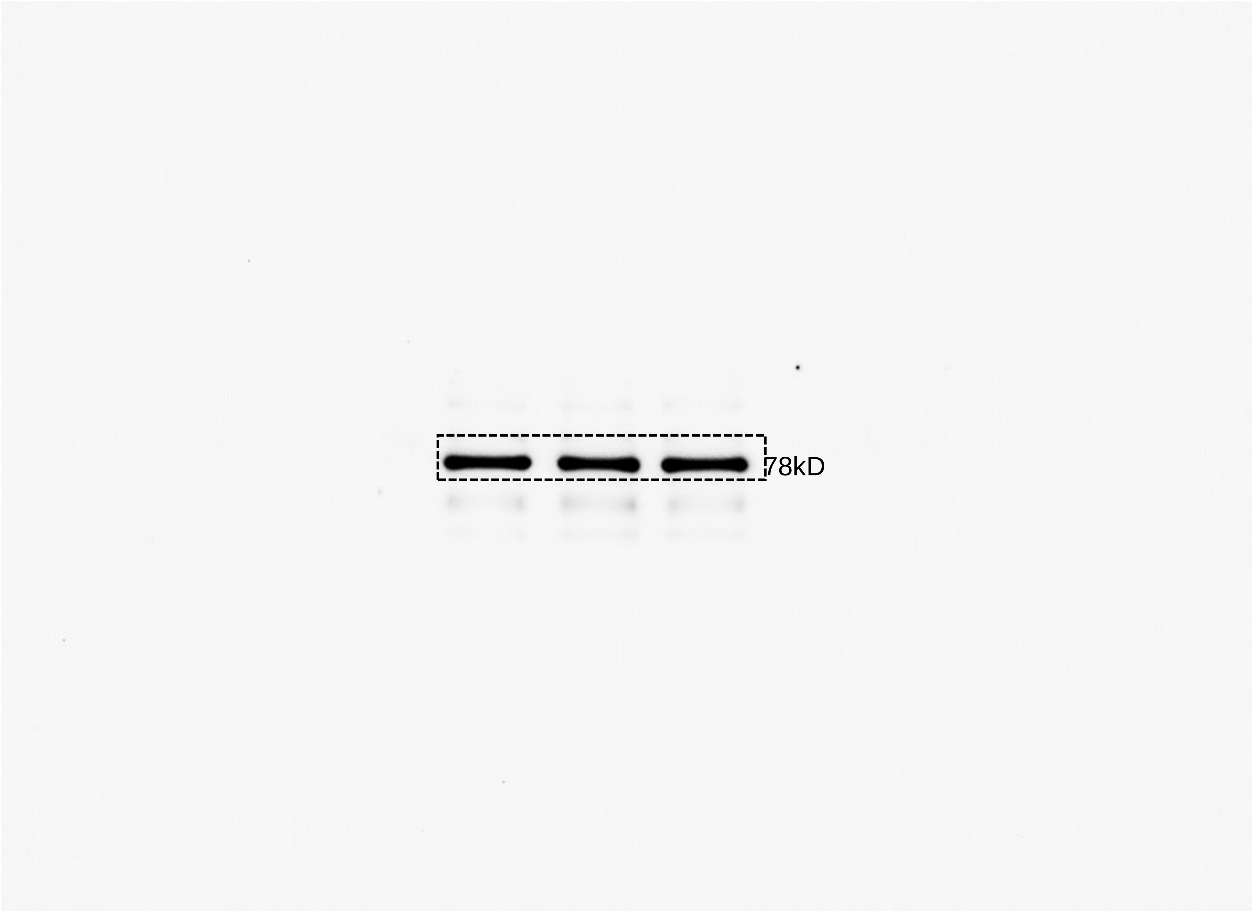

Figure S10 Full-length images of the immunoblots in Figure 4b. Black dot line boxes indicate the cropped images used in Figure 4b.

Figure S10

UVRAG

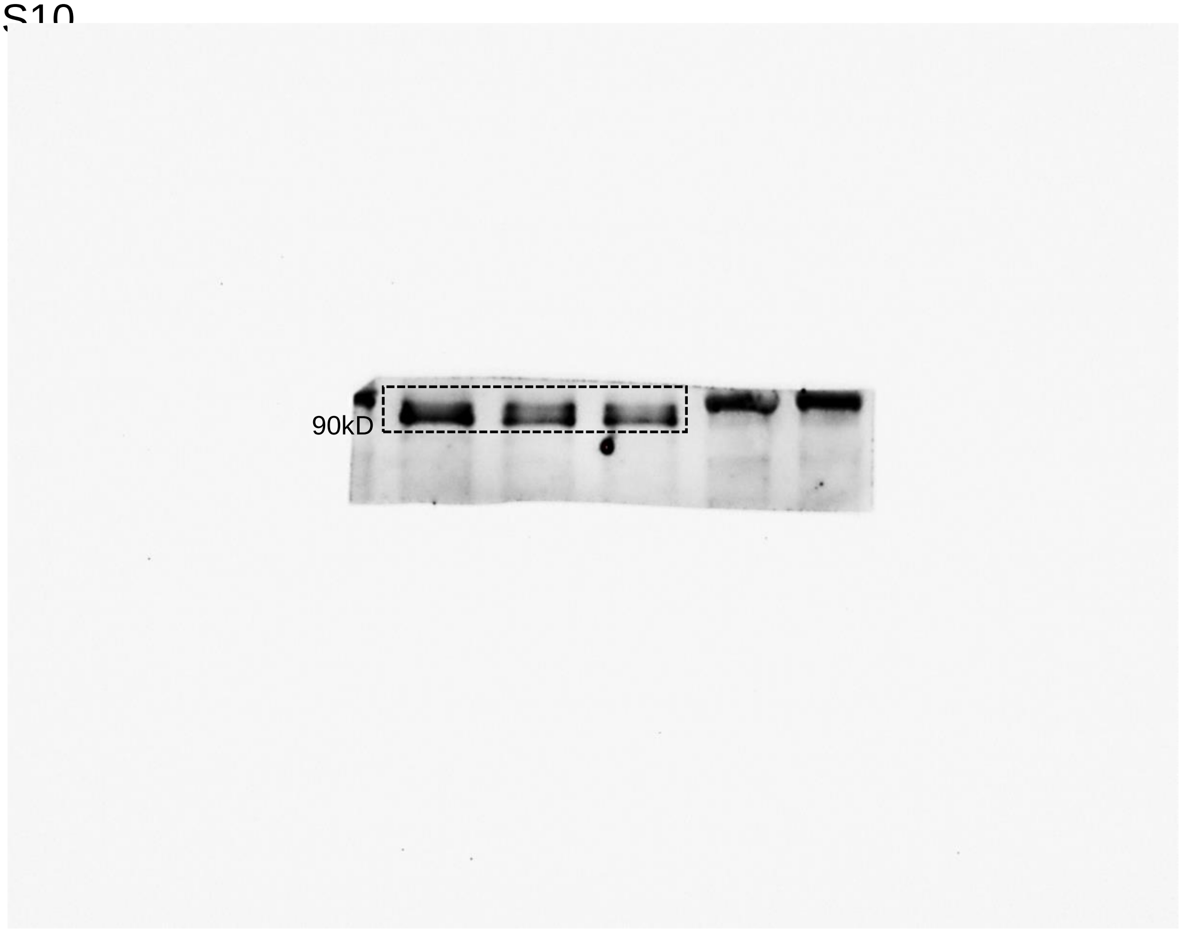

β-Actin

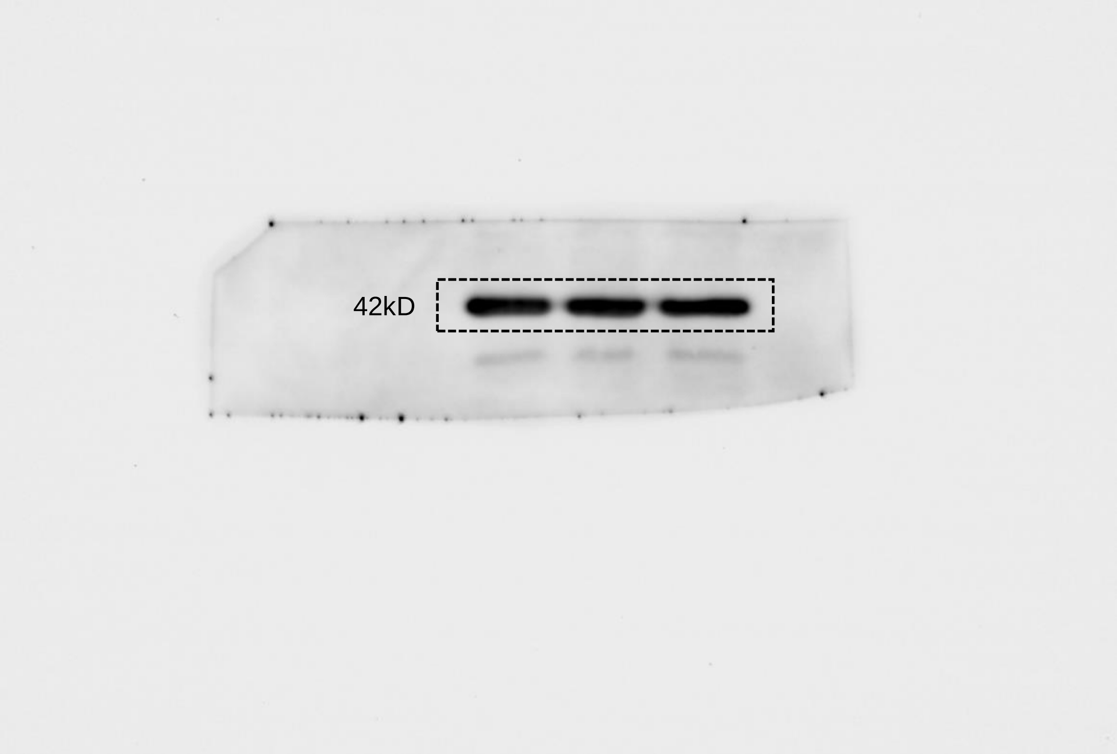

Figure S10 Full-length images of the immunoblots in Figure 4b. Black dot line boxes indicate the cropped images used in Figure 4b.

Figure S11

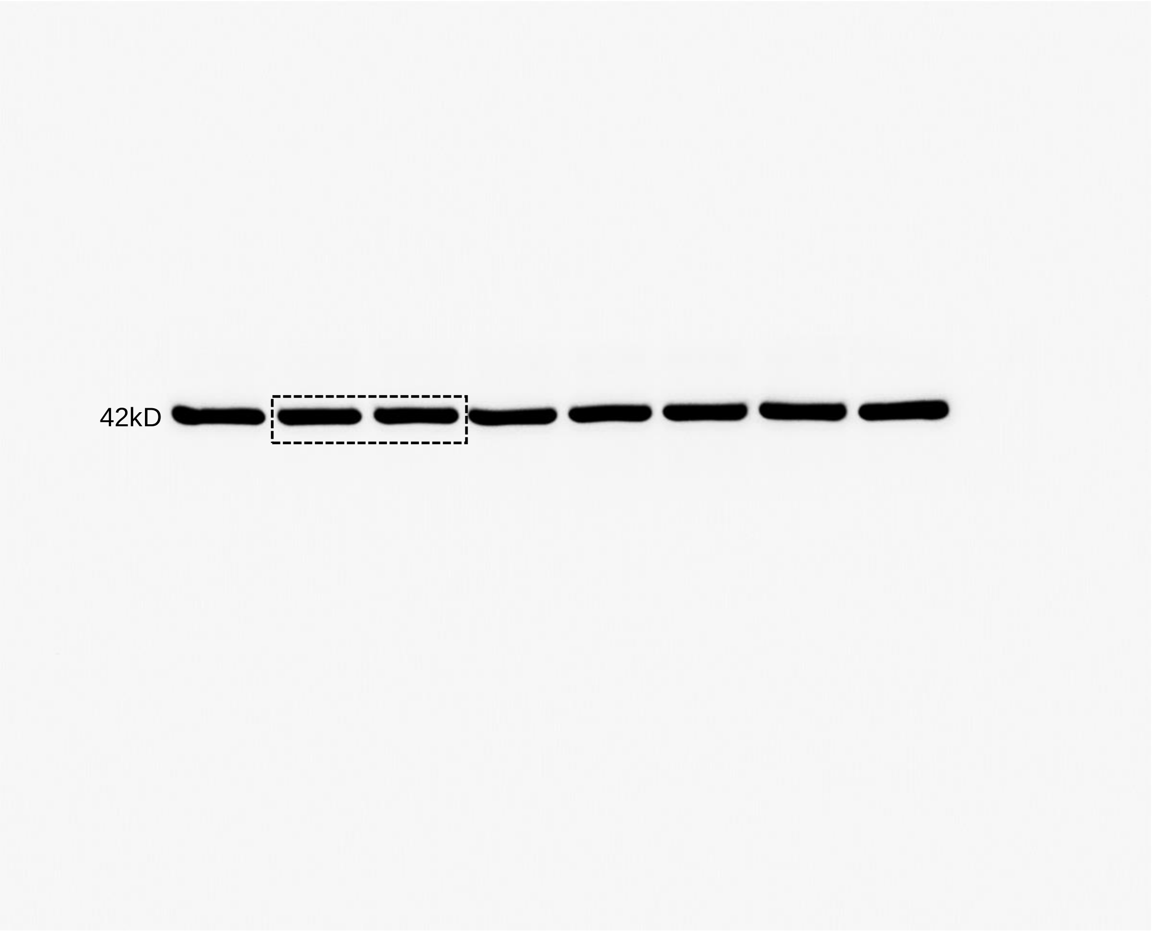

Figure S11 Full-length images of the immunoblots in Figure 4c. Black dot line boxes indicate the cropped images used in Figure 4c.

Figure S12

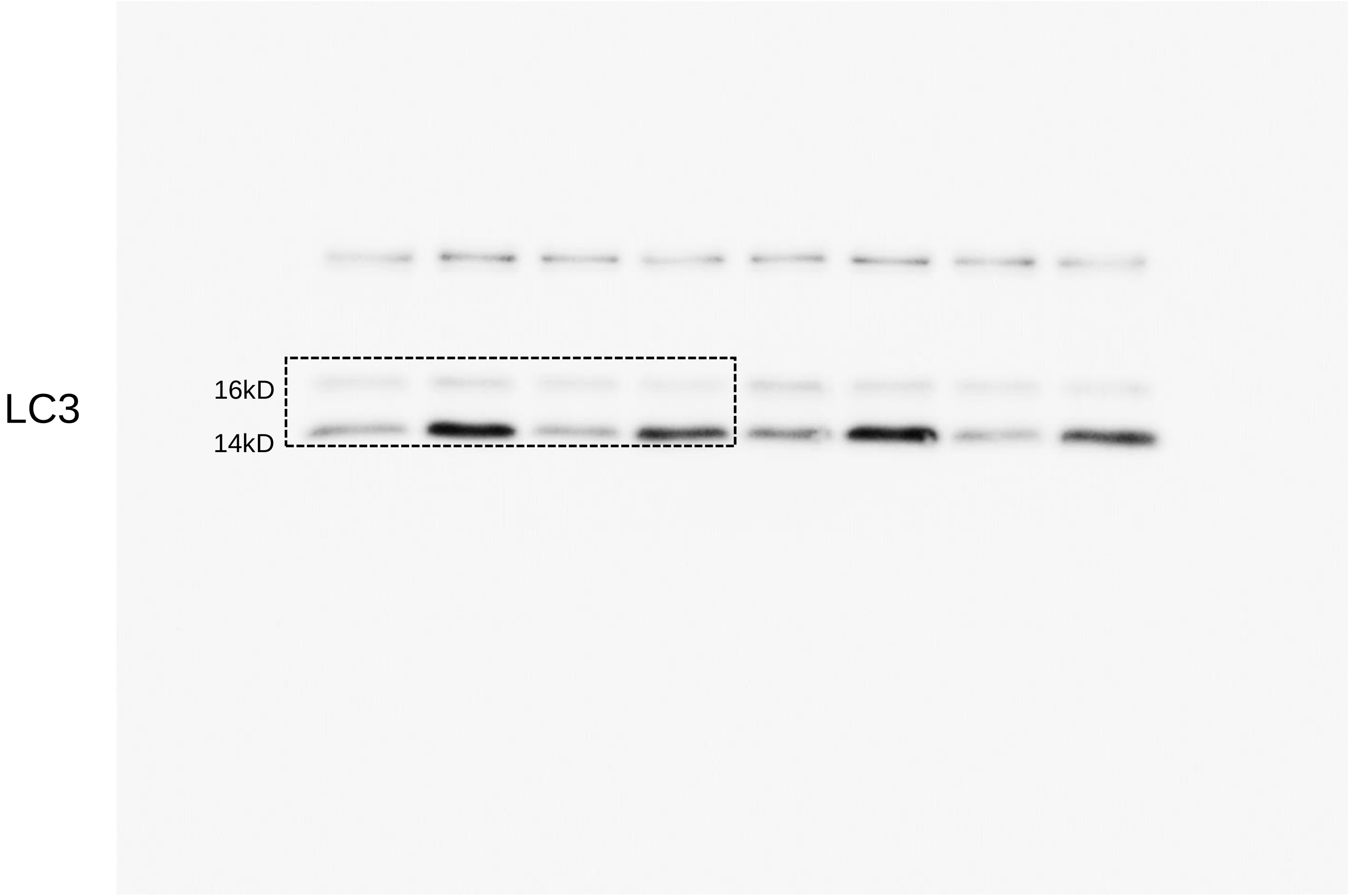

Figure S12 Full-length images of the immunoblots in Figure 4d. Black dot line boxes indicate the cropped images used in Figure 4d.

Figure S12

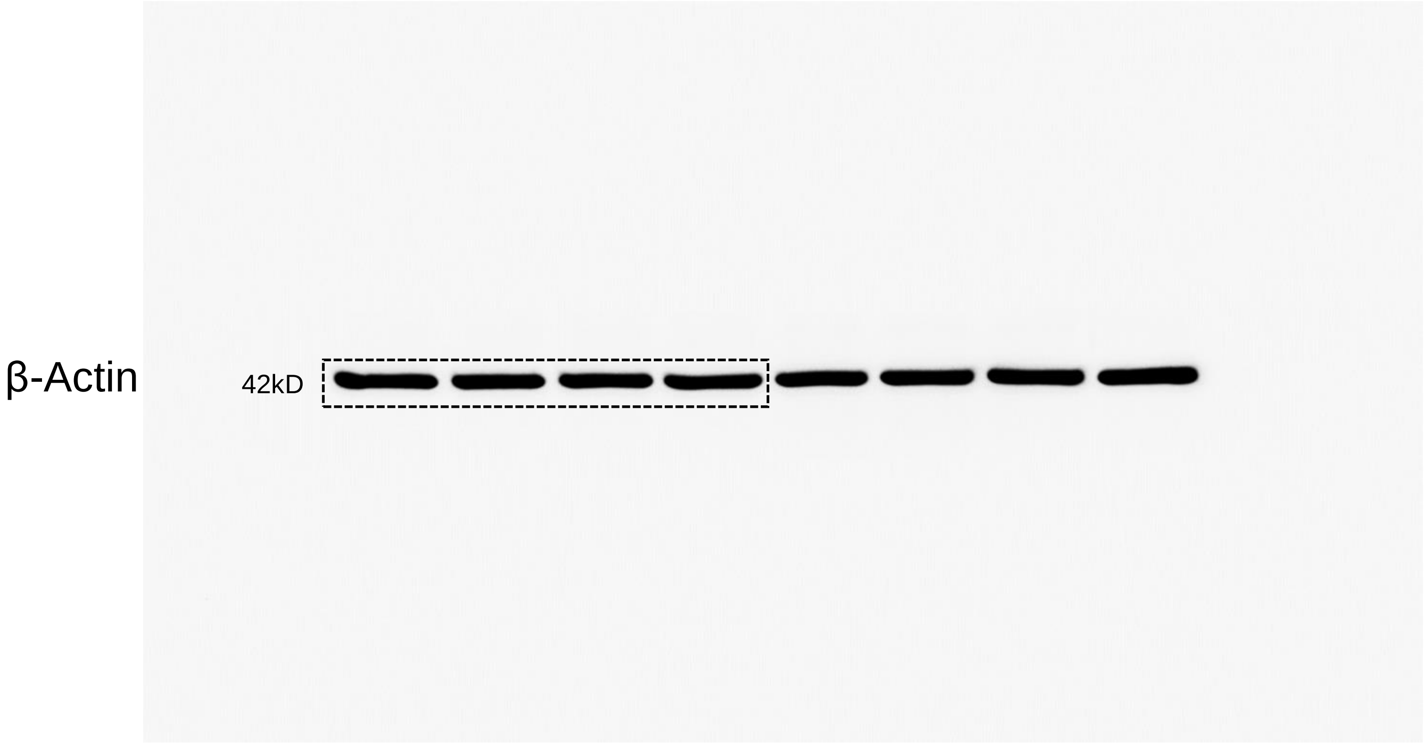

Figure S12 Full-length images of the immunoblots in Figure 4d. Black dot line boxes indicate the cropped images used in Figure 4d.
